# An Evolutionarily Conserved Enhancer Regulates *Bmp4* Expression in Developing Incisor and Limb Bud

**DOI:** 10.1371/journal.pone.0038568

**Published:** 2012-06-12

**Authors:** Dolrudee Jumlongras, Salil A. Lachke, Daniel J. O’Connell, Anton Aboukhalil, Xiao Li, Sung E. Choe, Joshua W. K. Ho, Annick Turbe-Doan, Erin A. Robertson, Bjorn R. Olsen, Martha L. Bulyk, Brad A. Amendt, Richard L. Maas

**Affiliations:** 1 Division of Genetics, Department of Medicine, Harvard Medical School, Brigham and Women’s Hospital, Boston Massachusetts, United States of America; 2 Department of Developmental Biology, Harvard School of Dental Medicine, Boston, Massachusetts, United States of America; 3 Department of Biological Sciences, Center for Bioinformatics and Computational Biology, University of Delaware, Newark, Delaware, United States of America; 4 Department of Aeronautics and Astronautics, Massachusetts Institute of Technology (MIT), Cambridge, Massachusetts, United States of America; 5 Institute of Biosciences and Technology, Texas A&M Health Science Center, Houston, Texas, United States of America; 6 Center for Biomedical Informatics, Harvard Medical School, Boston, Massachusetts, United States of America; 7 Department of Pathology, Harvard Medical School, Brigham and Women’s Hospital, Boston, Massachusetts, United States of America; 8 Harvard-MIT Division of Health Sciences and Technology, Harvard Medical School, Boston, Massachusetts, United States of America; University of Southern California, United States of America

## Abstract

To elucidate the transcriptional regulation of *Bmp4* expression during organogenesis, we used phylogenetic footprinting and transgenic reporter analyses to identify *Bmp4 cis*-regulatory modules (CRMs). These analyses identified a regulatory region located ∼46 kb upstream of the mouse *Bmp4* transcription start site that had previously been shown to direct expression in lateral plate mesoderm. We refined this regulatory region to a 396-bp minimal enhancer, and show that it recapitulates features of endogenous *Bmp4* expression in developing mandibular arch ectoderm and incisor epithelium during the initiation-stage of tooth development. In addition, this enhancer directs expression in the apical ectodermal ridge (AER) of the developing limb and in anterior and posterior limb mesenchyme. Transcript profiling of E11.5 mouse incisor dental lamina, together with protein binding microarray (PBM) analyses, allowed identification of a conserved DNA binding motif in the *Bmp4* enhancer for Pitx homeoproteins, which are also expressed in the developing mandibular and incisor epithelium. *In vitro* electrophoretic mobility shift assays (EMSA) and *in vivo* transgenic reporter mutational analyses revealed that this site supports Pitx binding and that the site is necessary to recapitulate aspects of endogenous *Bmp4* expression in developing craniofacial and limb tissues. Finally, Pitx2 chromatin immunoprecipitation (ChIP) demonstrated direct binding of Pitx2 to this *Bmp4* enhancer site in a dental epithelial cell line. These results establish a direct molecular regulatory link between Pitx family members and *Bmp4* gene expression in developing incisor epithelium.

## Introduction

Bmp4, a member of the TGF-β superfamily, is a secreted signaling molecule essential for embryogenesis [1]–[3]. It is expressed in a variety of tissues and organs throughout embryonic and postnatal life [4]. Evidence from mutations in mice and humans indicates that *Bmp4* regulates several developmental processes including patterning, proliferation, differentiation and apoptosis ([5]; http://omim.org/entry/112262).

The study of conditional *Bmp4* or *Bmp* receptor knockout alleles has shed light on the spatiotemporal functions of Bmp4 in epithelial-mesenchymal interactions during early craniofacial and limb morphogenesis [6]–[9]. For example, inactivation of *Bmp4* or *Bmp4r1a* in the facial primordia leads to isolated cleft lip or bilateral cleft lip and palate and deficient tooth development [10]. Furthermore, conditional deletion of *Bmp4* in distal mandibular arch ectoderm results in mandibular truncation and lack of incisors, indicating essential functions of epithelial Bmp4 in these ectodermal organs [7]. Bmp4 has also been implicated as one of the earliest signaling molecules secreted from the oral ectoderm that is capable of inducing dental mesenchymal genes that are necessary for tooth formation [7], [11]–[13].

*Bmp4* also plays an important role in regulating limb development, as suggested by its strong expression in the apical ectodermal ridge (AER), an important epithelial signaling center at the distal end of the limb bud, and in the anterior and posterior limb mesenchyme. Moreover, conditional inactivation of *Bmp4*, alone or in combination with other Bmps, or inactivation of its receptors, in limb bud AER or mesenchymal domains, has revealed roles in AER induction and maintenance respectively, as well as anteroposterior and dorsoventral limb patterning, and digit specification [9], [14]–[16], and chondrogenesis and osteogenesis [17]. In the developing limb, Bmp signaling has been shown to function in the context of an interconnected Bmp/Grem1 signaling module and a Shh/Grem1/Fgf feedback loop [18], while in the developing molar tooth, Bmp4 interacts with canonical Wnts as part of a feedback circuit that couples the development of the dental epithelium and mesenchyme [19].

As a step towards defining the gene regulatory networks (GRNs) that control *Bmp4* expression *in vivo*, we searched for *Bmp4* CRMs (*cis*-regulatory modules) using a transgenic reporter assay. Previously, Chandler et al. (2009) used a BAC reporter-based transgenic approach and identified a *Bmp4* lateral plate mesoderm (LPM) enhancer ∼46 kb upstream of the *Bmp4* transcription start site. We independently identified the same highly conserved, developmentally active *Bmp4* regulatory region, but have extended the prior characterization by Chandler et al. (2009) of a 4.3 kb CRM and of a smaller 467 bp subregion to reveal several important new attributes [1].

We refined this CRM to an essential 396-bp minimal enhancer that confers reporter gene expression in developing distal mandibular and incisor epithelium and the limb bud, tissues that require Bmp4 for proper morphogenesis. We also used TF (transcription factor) DNA binding specificity motif data from the UniPROBE database [20], [21] to analyze the *Bmp4* enhancer and identified a high-affinity binding sequence for Pitx homeobox TFs, which are strongly expressed in the developing mandible, tooth and limb, and which have been implicated in human and mouse odontogenic defects and lower limb malformations [22]–[24]. *Pitx1* may activate gene expression in dental epithelium [25], while *Pitx2* null mutants exhibit an early stage arrest in tooth development [26], [27]. In hindlimb development, *Pitx1^−/−^* and *Pitx2^−/−^* double mutants exhibit altered signaling molecule expression in the AER, which is proposed to account for the proximal limb reduction defect in these mutants [28]. However, the molecular regulatory relationship between *Pitx1, Pitx2* and *Bmp4* remains unclear. We show here that Pitx homeoprotein family members bind a specific site in the *Bmp4* incisor epithelium limb bud (“IE/LB”) enhancer that is necessary for its activity *in vivo*. These results define a minimal, highly conserved *Bmp4* enhancer and identify Pitx homeoproteins as key TFs that regulate its embryonic expression.

## Results

### Identification of Putative *Bmp4* CRMs by Phylogenetic Footprinting

To identify candidate *Bmp4* enhancers, we conducted a phylogenetic footprinting analysis [29]–[31] on the genomic region surrounding the mouse *Bmp4* gene. The mouse *Bmp4* transcription unit is located on chromosome 14qC1, and spans ∼7 kb with five exons and two alternative TATA-less promoters [32], [33]. We focused our search on a 159 kb region consisting of two 76 kb non-genic regions upstream and downstream of *Bmp4*, and the 7 kb *Bmp4* transcription unit itself. We compared this 159 kb mouse genomic sequence, obtained from the UCSC genome database and devoid of other known genes and ESTs, with homologous genomic sequences of human and pufferfish (*Takifugu rubripes*, or *Fugu*) using the local alignment program BLASTZ which displays homology as blocks of sequence conservation [34], [35]. The *Fugu* sequence, although distantly related, was used in the analysis based on the assumption that non-coding sequences that have tolerated selective pressure for hundreds of million years of evolution are likely to be functionally significant and to play important roles in gene regulation [36]–[38]. While not all morphogenetic programs in which *Bmp4* plays a critical role (*e.g.,* limb and tooth development) are likely to be fully conserved in fish, we reasoned that certain core regulatory sequences might be. Moreover, we hypothesized that the compactness of the *Fugu* genome could further filter the relatively high degree of conservation between human and mouse, thereby prioritizing putative regulatory regions for further analysis.

**Figure 1:**
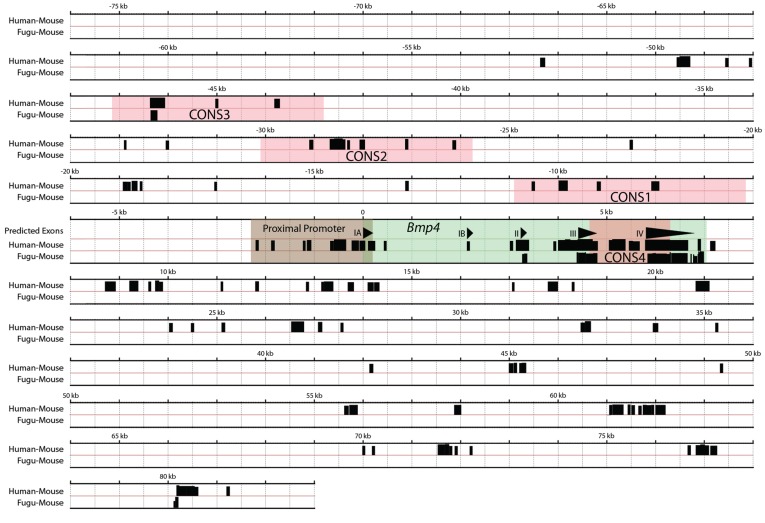
Human, mouse and fugu sequence conservation surrounding the *Bmp4* gene. Homologous genomic pair-wise alignments between human and mouse (Human-Mouse) and *Fugu* and mouse (Fugu-Mouse) were generated using BLASTZ. Genomic sequence of 159 kb surrounding the mouse *Bmp4* gene (76 kb 5′ of the *Bmp4* transcriptional start site and 83 kb 3′ of it) was used as reference. The *Bmp4* gene (green) consists of 4 exons as (triangles denote IA, IB, II, III and IV) with the 2.4 kb proximal *Bmp4*IA promoter region (brown) upstream of exon IA. Transcriptional direction is designated by the exon triangles and genomic positions upstream and downstream of the transcriptional start site (position 0) are denoted in (−) and (+) numbers, respectively. Human-Mouse and Fugu-Mouse homologies of >75% identity over 50 bp regions are shown as blocks of sequence conservation. CONS1, CONS2, CONS3 and CONS4 (pink) encompass clusters of conservation blocks. Note: Two blocks of Fugu-Mouse sequence conservation are located 46 kb upstream and 80 kb downstream of the *Bmp4* transcription start site, respectively. The 46 kb upstream Fugu-Mouse conservation block is embedded within the CONS3 region.

The BLASTZ alignment identified several discrete blocks of sequence conservation between mouse and human, using parameters of >75% identity over >50 bp, which have similar stringency to parameters previously used for identification of functional mammalian regulatory elements [39], [40]. In addition to highly conserved sequences representing *Bmp4* exons IA, IB, II, III and IV, 87 blocks of sequence conservation (31 blocks located 5′ and 56 blocks located 3′ to the *Bmp4* transcription start site) between mouse and human were identified and considered as candidate *Bmp4* regulatory sequences (Figure 1). While several of these blocks cover long genomic regions, others are smaller and reside in clusters. Previous studies indicate that conserved non-coding regions of developmental genes frequently cluster together [38], [41]. Therefore, we grouped closely residing conservation blocks of 200 bp or longer into larger conserved or “CONS” regions. Based on human-mouse sequence conservation analyses, we selected four highly conserved regions and their immediate flanking sequences upstream and within the *Bmp4* gene as high priority candidate regulatory sequences. These sequences were designated as CONS1, CONS2, CONS3 and CONS4, with their respective 5′ boundaries located 11 kb, 30 kb, and 47 kb upstream, and 4.6 kb downstream of the *Bmp4* transcription start site; the latter region resides within an intron in the *Bmp4* gene (Figure 1).

When the orthologous *Fugu* genomic sequence was included in the analysis, only two blocks of conservation, located ∼46 kb 5′ upstream and ∼80 kb 3′ downstream of the mouse *Bmp4* transcription start site, were conserved in all three genomes. Both blocks are embedded within larger regions of human-mouse conservation, and the ∼46 kb upstream block is located within the CONS3 sequence, but only the ∼46 kb upstream block contained conserved regions exceeding our 200 bp cut-off (Figure 1). To verify that these conserved regions represented non-coding sequences, we performed a BLAST search and re-confirmed that they did not match any known coding regions, mRNAs or ESTs. We refer to the previously described 2.4 kb promoter region located upstream of the *Bmp4* transcription start site as the proximal promoter, to distinguish it from a second distal promoter located within intron 1 [42], and from other regulatory elements described in the present work (Figure 1). The 2.4 kb proximal *Bmp4* promoter also shows high human-mouse conservation (Figure 1) and that in transgenic mice, this region in isolation has been shown to drive *Bmp4* expression in epithelial-derived ameloblasts and hair shaft keratinocytes and matrix [43], [44]. In addition, the mammalian proximal promoter region does not show conservation with *Fugu* under the stringency used in this analysis (Figure 1). Collectively, these results suggest that other more distant elements regulate the full repertoire of *Bmp4* developmental expression.

### CONS3 Recapitulates *Bmp4* Expression in Multiple Tissues during Development

**Figure 2:**
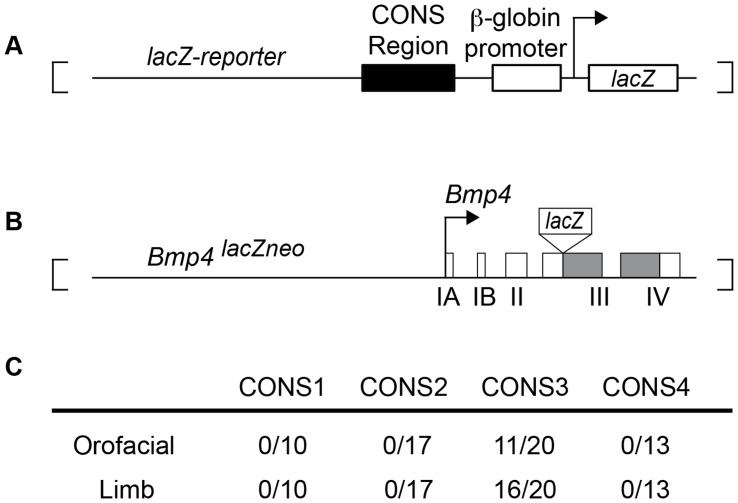
Conserved region driven β-galactosidase activity in the orofacial region and limb. (**A**) Schematic of the transgenic reporter construct used in transient transgenic analyses. The black rectangle denotes the location of CONS region insertions upstream of the human beta globin promoter in pGLKS (see Materials and Methods). (**B**) Schematic of the *Bmp4^lacZneo^* allele [45] (white boxes, exons; gray boxes, coding regions). (**C**) Number of transient transgenic embryos with various CONS derivative constructs that supported β-galactosidase expression in limb or orofacial tissue.

To determine if these candidate regulatory regions harbored transcriptional regulatory activity, we tested the ability of each individual CONS region to drive expression of a *lacZ* reporter gene in transgenic mouse embryos at embryonic day (E) 11.5 and compared them to E11.5 embryos heterozygous for a *Bmp4^lacZneo^* reporter allele [45] (Figure 2A, B). Transient transgenic analysis of CONS1 (n = 10), which spans 4.7 kb and includes 4 highly conserved blocks, and CONS4 (n = 13), which extends approximately 1.7 kb over the intronic region between exons III and IV, showed no reporter activity in any of the known *Bmp4* expression domains at E11.5 (Figure 1 and Figure 2C). Transgenic embryos carrying the 4.3 kb CONS2 transgene, which consists of six conserved blocks, showed consistent expression in the developing forebrain (n = 17). Although not a domain where endogenous *Bmp4* is normally expressed, this validates the functional competence of the CONS2 construct. It is also possible that CONS1, 2 ands 4 are expressed in the adult animal, or at developmental times different from the E11.5 time point assayed here.

**Figure 3:**
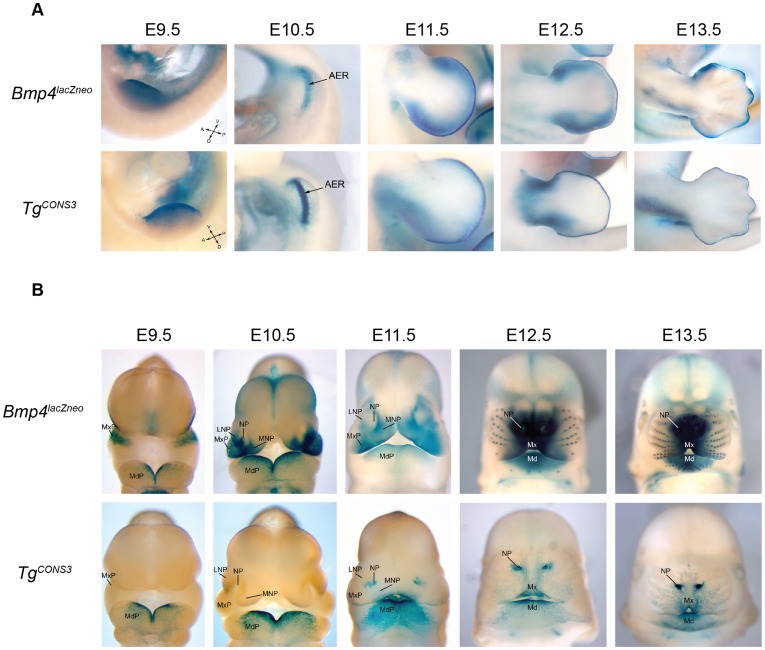
*Bmp4^lacZneo^* and *Tg^CONS3^* β-galactosidase activity in the limb and orofacial region. (**A**) Lateral views of developing fore- or hindlimbs of *Bmp4^lacZneo^* control mouse embryos (*upper row*). *Tg^CONS3^* transgenic embryos from permanent transgenic line TL3459 (*lower* row). The CONS3 transgene expression largely recapitulates endogenous *Bmp4* expression in the AER from E9.5–13.5. (**B**) Frontal views of the developing head of *Bmp4^lacZneo^* and *Tg^CONS3^* transgenic embryos from permanent line TL3459. From E9.5 to E11.0, *Bmp4* is expressed in the epithelium overlying the distal first branchial arch, maxillary and mandibular processes and medial and lateral nasal processes. At these stages, the CONS3 enhancer recapitulates endogenous expression in the ectoderm overlying the distal part of the first branchial arch at E9.5 and the mandibular process at E10.5. At E12.5, endogenous *Bmp4* expression shifts to the mesenchyme, while CONS3 expression persists in the epithelium overlying the mandibular arch and premaxilla, including incisor tooth germs. At E12.5 and 13.5, endogenous *Bmp4* is concentrated in the mesenchyme of the midface including the whisker follicles. CONS3 transgene expression persists in the epithelium that overlies the mandible, pre-maxilla and nasal pits, and fails to shift to the underlying mesenchyme. Abbreviations: MxP, maxillary process; MdP, mandibular process; MNP, medial nasal process; LNP, lateral nasal process; NP, nasal pit; Mx, maxilla; Md, mandible.

In contrast, transient transgenic analysis of the 4.3 kb CONS3 region, which includes one of the two human-mouse-*Fugu* conserved blocks and has 94% overall human-mouse homology, revealed that a subset (n = 11/20) of embryos exhibited transgene expression in the oral epithelium overlying the maxillary process and mandibular arch, a pattern similar to that of endogenous *Bmp4* (Figure 2C; Figure 3). The epithelial incisor dental lamina placodes also exhibited transgene expression in a pattern similar to that of endogenous *Bmp4* (Figure S1). We also detected transgene expression specific to the limb bud apical ectodermal ridge (AER), and in forelimb and hindlimb posterior mesenchyme (n = 16/20), with weaker expression in anterior limb mesenchyme, similar to that of endogenous *Bmp4* (Figure 3). Transgene expression was also observed in the proximal limb where endogenous *Bmp4* is normally expressed (Figure 2C, Figure 3). Thus, CONS3 contains *cis*-regulatory sequences that are capable of acting on a heterologous beta-globin promoter to direct gene expression to the orofacial region, the AER, and limb bud mesenchyme in a pattern similar to that of endogenous *Bmp4*.

*Bmp4* expression in the developing teeth and craniofacial region is dynamic [46], particularly at E11.5 when expression begins to shift from the epithelium to the underlying mesenchyme. Therefore, we generated permanent 4.3 kb CONS3 transgenic lines that allowed us to analyze the spatiotemporal activity of the CONS3 enhancer in multiple embryos at different developmental stages. Three transgenic male founders, TL3459, BB3482 and KR3495, were established and crossed with wild type females to produce several litters of F1 embryos, which were analyzed for *lacZ* activity from E9.5 to E13.5. The expression of the CONS3 transgene during limb bud development in all three permanent lines exhibited reporter activity in endogenous *Bmp4* expression domains at these stages (Figure 3A).

While CONS3 appears to control most of the major spatiotemporal aspects of *Bmp4* expression in the developing limb, its expression in the mid-facial region is more complex. At E9.5, the CONS3 transgene is expressed in the ectoderm overlying the distal region of the developing mandible of the first branchial arch, a pattern similar to that in *Bmp4^lacZneo^* embryos (Figure 3B). From E9.5 to E11.0, transgene expression recapitulates the endogenous pattern and is maintained in the distal mandibular arch epithelium and in the dental lamina of the developing incisors, with weaker expression in the medial and lateral nasal processes (Figure 3B). Notably, however, at E11.5 when endogenous *Bmp4* expression begins to shift to the underlying mesenchyme of the maxillary and mandibular arches, CONS3 enhancer activity persists in the epithelium overlying the pre-maxilla, nasal pits, distal mandible, and in the incisor epithelial bud, until at least E13.5 (Figure 3B). This indicates that while CONS3 contains *cis*-regulatory elements that drive reporter gene expression in mandibular and incisor dental epithelium, it lacks the elements necessary for *Bmp4* expression in the dental mesenchyme. In addition, the CONS3 reporter seems to escape the normal downregulation of epithelial expression that accompanies endogenous *Bmp4* expression (Figure S2).

### Refinement of the *Bmp4* Enhancer to a Minimal 396-bp Conserved Region

**Figure 4:**
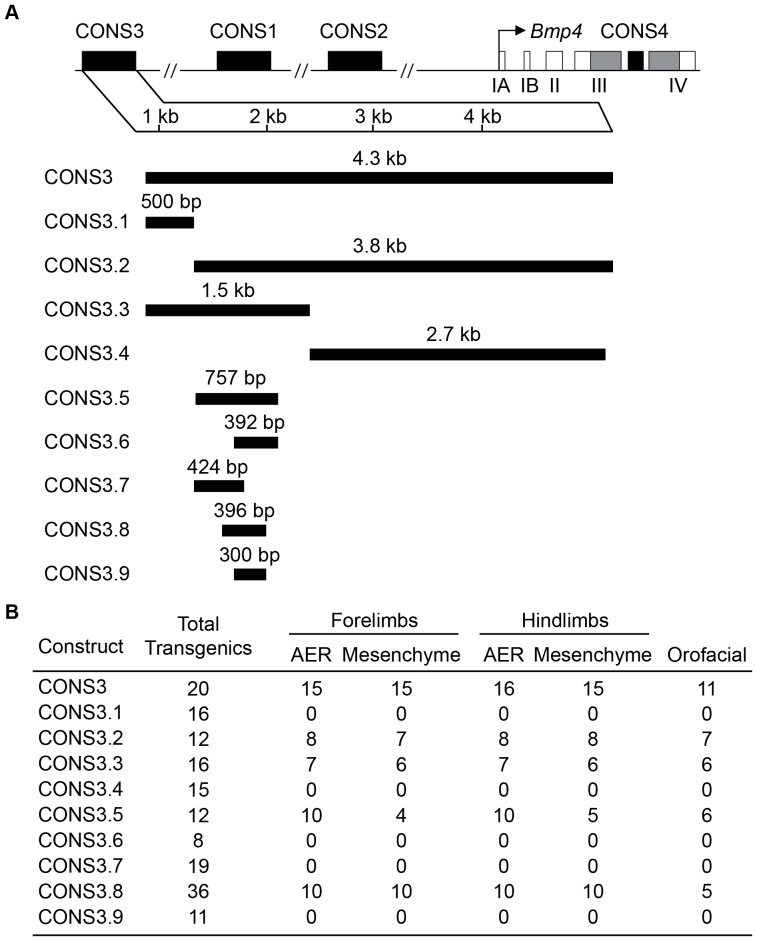
Deletion analysis of the *Bmp4* enhancer CONS3. (**A**) Schematic of the four regions (CONS1-4; black boxes), each containing multiple human-mouse sequence conservation blocks, tested in a transient transgenic mouse reporter assay in the same reporter shown in Figure 2. The CONS3 enhancer was systemically narrowed down to 758 bp region, CONS3.5, which contains the Fugu-Mouse upstream conservation block, and then to a minimal 396 bp minimal enhancer. (**B**) The number of E11.5 transgenic mouse embryos showing endogenous *Bmp4* orofacial and limb expression (in the AER and mesenchyme region of fore- and hindlimbs) is reported over the total number of transgenic embryos analyzed (No. Stained/No. Tg).

To define the minimal CONS3 sequence sufficient for limb and mandibular enhancer activity, we performed a series of deletion experiments. The deletion constructs, designated CONS3.1 to CONS3.9, were screened for *lacZ* activity in E11.5 transient transgenic embryos to determine whether the remaining sequence conferred reporter expression in the same tissue domains as intact CONS3 and endogenous *Bmp4* (Figure 2B and Figure 4A, B). CONS3.1, which covers 500-bp of the 5′ end of CONS3, did not confer any activity of the reporter gene; however, CONS3.2, which spans 3.8 kb of the 3′ portion of CONS3 reproduced expression of the entire 4.3 kb CONS3 construct (Figure 4A, B). The 1.5 kb CONS3.3 fragment, a subset of CONS3, also retained CONS3 activity (Figure 4A, B). We then generated and tested CONS3.5, which covers 757 bp with the highest sequence conservation within CONS3.3, and found that it also retained enhancer activity similar to that of CONS3 (Figure 4A, B). We also noted consistent ectopic expression in the midbrain of CONS3.5 transgenic embryos (data not shown), which suggests that sequences outside CONS3.5 and within CONS3.3 may possess repressive activity for *Bmp4* reporter transgene expression in midbrain. Lastly, the CONS3.5 fragment was tested in an orientation opposite to that of the endogenous locus, which establishes its orientation independence.

We further investigated CONS3.5 activity during incisor morphogenesis. At E14.0, CONS3.5 expression was maintained in cap-stage dental epithelium, but absent from the dental mesenchyme and enamel knot; the latter is an epithelial signaling center that expresses endogenous *Bmp4* and controls tooth cusp patterning [47]. Endogenous *Bmp4* is normally expressed in the enamel knot, and downregulated in the surrounding dental epithelium. Thus, in contrast to its activity in mandibular ectoderm and lamina stage incisor epithelium, which is faithful to endogenous *Bmp4* expression, at the cap-stage CONS3.5 directs epithelial expression in the developing incisor in a complementary pattern to that of endogenous *Bmp4* (Figure S2). While beta-galactosidase perdurance cannot be entirely excluded, it seems much more likely that genomic regions outside of CONS3.5 are required for the repression of *Bmp4* in the dental epithelium after the lamina stage. Moreover, the results indicate that sequences outside CONS3.5 are required for the activation of *Bmp4* expression in the dental mesenchyme and enamel knot.

To further define the minimal genomic region required for *Bmp4* enhancer activity, we subcloned overlapping 3′ and 5′ halves of CONS3.5, designated CONS3.6 (392-bp) and CONS3.7 (424-bp) respectively, into reporter vectors and tested their ability to confer expression. Although a few CONS3.7 embryos exhibited reporter gene activity in the proximal limb, neither construct gave detectable reporter activity in the AER, mandibular arch or incisor dental lamina (CONS3.6, CONS3.7); hence these were scored as negative (Figure 4A, B). In contrast, CONS3.8, a 396-bp sequence from the approximate mid-region of CONS3.5 that overlaps CONS3.6 and CONS3.7, exhibited reporter activity in the same domains as CONS3 (Figure 4A, B), albeit at lower levels. Further deletion of the 5′ and 3′ ends of CONS3.8 to generate a 300-bp CONS3.9 construct yielded no enhancer activity in any of the CONS3 expression domains (Figure 4A, B).

Thus, the 396-bp CONS3.8 sequence represents the minimal enhancer element necessary for *Bmp4* expression in the AER and limb bud mesenchymal domains, and in mandibular arch and incisor dental lamina epithelia. Although its full expression properties remain to be explored, we have provisionally denoted this *cis*-regulatory module the *Bmp4* incisor epithelium/limb bud, or “IE/LB” enhancer. In addition, since the entire 396 bp IE/LB enhancer is contained within the 467 bp LPM (lateral plate mesoderm) enhancer previously characterized by Chandler and coworkers [1], the 467-bp sequence likely functions as a composite IE/LB and LPM, or “IE/LB/LPM” enhancer, although this was not tested directly by comparing the behavior of both the 396 and 467 bp sequences at the time point at which LPM activity was detected [1].

### The Minimal *Bmp4* CONS3.8 Enhancer Contains a Conserved *Pitx* and *Msx* Binding Motif

To identify potential direct regulators of the *Bmp4* enhancer, we searched the UniPROBE database (http://thebrain.bwh.harvard.edu/uniprobe/) for putative transcription factor (TF) binding sites within the minimal 396-bp *Bmp4* enhancer (CONS3.8) sequence. To further restrict the list of candidate TF regulators, we generated and analyzed microarray gene expression datasets from tissues with CONS3.8 enhancer activity. Epithelial gene expression analysis, using laser capture microdissection (LCM) of E11.5 mouse incisor tooth germs (Figure S3), and previously published E12.5 fore- and hindlimb microarray datasets, were used in combination to identify genes whose transcripts were consistently expressed (*i.e.,* ≥ 2 of 3 replicates). This list of expressed genes was then intersected with the list of TF families having conserved putative binding sites in the minimal CONS3.8 *Bmp4* enhancer (see Materials and Methods). In summary, fourteen TF families, which included the Pitx and Msx TF families, exhibited conserved potential binding sites in the minimal CONS3.8 *Bmp4* enhancer (Table S1).

### The CONS3.8 Enhancer Supports Pitx and Msx Protein Binding *in vitro*

**Figure 5:**
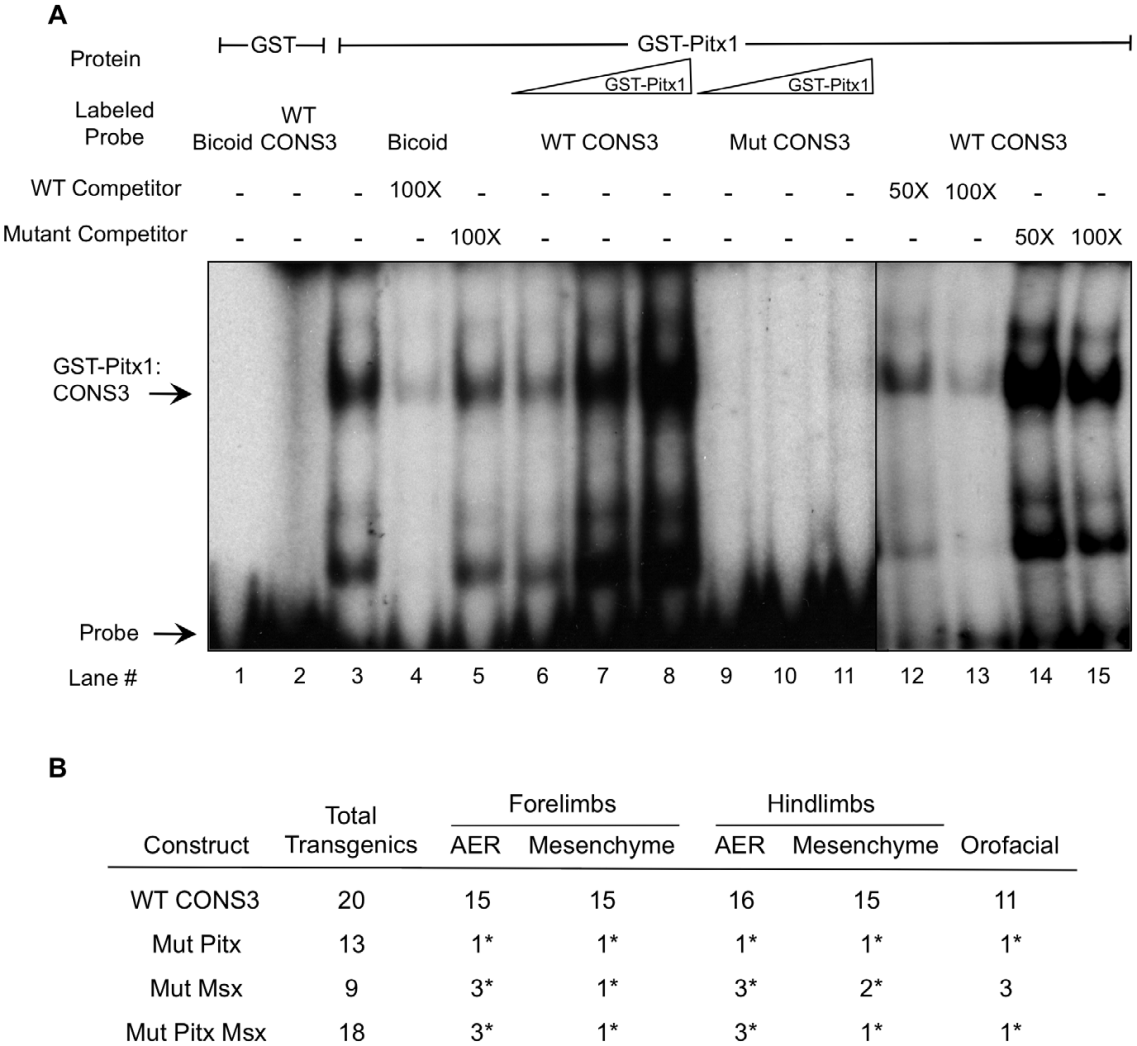
Pitx binds to the minimal *Bmp4* enhancer CRM *in vitro* and the Pitx binding site is necessary for enhancer activity. (**A**) Electrophoretic Mobility Shift Assay (EMSA) exhibits robust binding of Pitx1 protein to both a positive control *bicoid* DNA sequence and to the consensus Pitx1/2 binding site in with a 25 bp probe sequence in the CONS3.8 sequence. Competition with specific or non-specific unlabelled probes indicates sequence-specific binding of GST:Pitx1 fusion protein to the consensus Pitx1/2 DNA binding motif. (**B**) Number of transient transgenic embryos that supported β-galactosidase expression in forelimbs, hindlimbs, or orofacial tissue. Asterisk indicates statistically significant differences (p<0.05) compared to wild-type CONS3 by Fisher’s exact test.

Among these candidates, we chose to focus only on those TFs previously implicated in limb, tooth or mandible development [18], [19], [24], [48]. The above analyses yielded strong candidate binding sites for the Pitx (5′-TAATCC-3′) and Msx (5′-GTAATTG-3′) TF families within the minimal 396-bp enhancer (Figure S4). We next performed Electrophoretic Mobility Shift Assays (EMSA) to determine whether Pitx or Msx proteins can specifically bind to their respective predicted binding sites in the CONS3.8 enhancer. We generated full-length Pitx1 and Msx2 GST-fusion proteins and incubated them with 25-mer sequences taken from within CONS3.8 that encompassed either the Pitx or Msx binding sites, and compared DNA-protein complex formation to that with probes consisting of canonical DNA recognition sequences for each protein [49], [50]. Both Pitx1 and Msx2 proteins specifically bound the wild type *Bmp4* enhancer (WTPitxBSCONS3 or WTMsxBSCONS3) and their canonical sequences (Bicoid or MBS, respectively) (Figure 5A and Figure S5; lanes 3, 6–8). In addition, these complexes were specifically competed by excess (50-fold or 100-fold) unlabeled wild type *Bmp4* enhancer oligonucleotide competitor (WTPitxBSCONS3 or WTMsxBSCONS3) (Figure 5A and Figure S5; lanes 12–15), confirming the binding specificity of each TF with its respective binding site.

To ascertain the necessity of these intact DNA sites for protein binding, we introduced mutations into each site that were predicted to abolish binding (see Materials and Methods). As expected, EMSA demonstrated that the proteins could not bind the respective mutant oligomers (Figure 5A and Figure S5). In addition, unlike their wild type counterparts, unlabeled mutant oligomers at 50- to 100-fold excess did not compete with wild type oligomer binding. Thus, the CONS3.8 396-bp *Bmp4* minimal enhancer sequence contains specific, high affinity Pitx and Msx binding sites that support binding of these proteins *in vitro*. This is also in agreement with the prediction from PBM analysis, in which both Pitx and Msx displayed strong sequence preference for their respective putative binding sites (PBM enrichment (E) score >0.45).

### CONS3 Enhancer Activity *in vivo* Requires an Intact Pitx Motif

To test the functionality of the Pitx and Msx binding sites in CONS3 *in vivo*, we assayed the activity of CONS3 enhancer sequences that contained clustered point mutations in the respective binding sites in transient transgenic mice. The introduced mutations fulfilled the criteria of completely abolishing TF binding in EMSAs, and insofar as could be determined, did not generate an adventitious site that could bind other TFs.

Transient transgenic mice carrying *MutPitxCONS3* and *MutMsxCONS3* reporter transgenes with mutations in Pitx and Msx binding sites respectively, displayed significantly reduced reporter activity compared to wild type (Figure 5B and Figure S6). When each of the CONS3 limb and orofacial expression domains was examined for β-galactosidase activity individually, only a minority of mutant embryos exhibited expression when compared to wild type CONS3 embryos (Figure 5B and Figure S6). Moreover, when transgenic embryos were stringently scored for expression in all CONS3 expression domains, only one embryo for each mutant construct exhibited *lacZ* expression in all domains. This reduction in mutant CONS3 enhancer activity is statistically significant (*p*<0.05, Fisher’s exact test) when compared to the 55% of wild type CONS3 transgenics that were *lacZ*-positive in all domains (Figure 5B and Figure S6).

To test whether the Pitx and Msx binding sites might cooperate to activate the CONS3 enhancer, we engineered a construct, *MutPitxMsxCONS3,* which carried mutation in both Pitx and Msx binding sites and assayed it in transient transgenic mouse embryos at E11.5. This analysis revealed that a few of *MutPitxMsxCONS3* transgenic embryos (n = 3/18) exhibited very weak and spotty expression in the AER and limb mesenchyme and only 1 of these embryos showed faint transgene expression in the oral epithelium (n = 1/18) (Figure 5B and Figure S6). Thus, since the *MutPitxMsxCONS3* transgene retains low but detectable enhancer activity similar to that of the *MutPitxCONS3* and *MutMsxCONS3* transgenes, the Pitx and Msx binding sites do not appear to cooperate synergistically. Instead, each motif appears to function largely independently to activate the CONS3 enhancer *in vivo*.

### Pitx2 Directly Binds the *Bmp4* Enhancer *in vivo*

**Figure 6:**
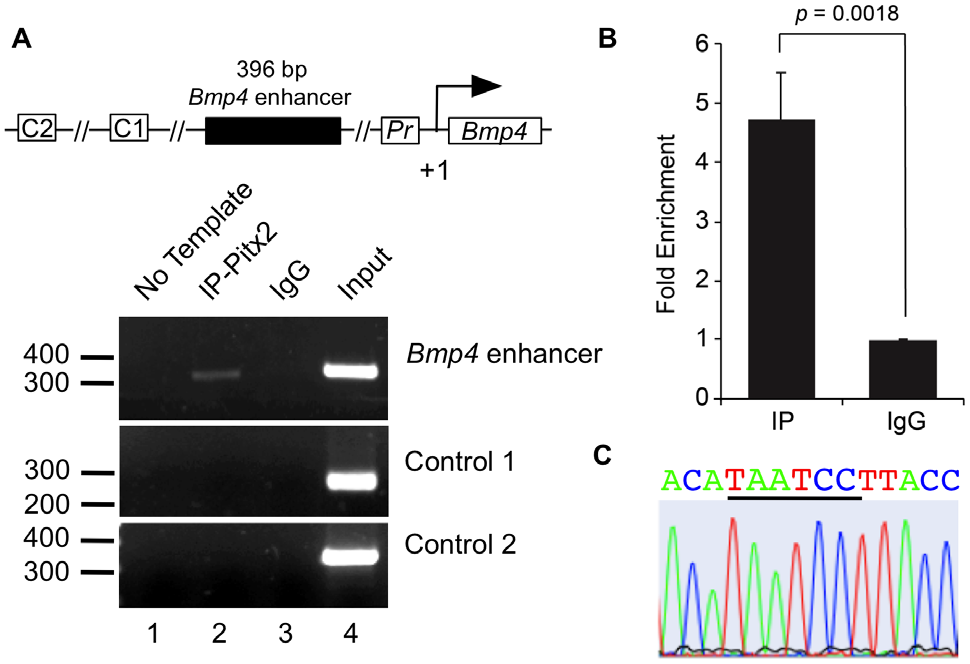
Pitx binds the minimal *Bmp4* enhancer *in vivo*. (**A**) Schematic representation of the ChIP assay design. The 396-bp *Bmp4* CRM containing the Pitx1/2 binding site is shown along with two control regions, C1 and C2. C1 and C2 are located upstream of the *Bmp4* transcription start site and do not contain Pitx1/2 binding sites (see Materials and Methods). The transcriptional start site (TSS) is marked as +1 and Pr is the *Bmp4* proximal promoter. Chromatin Immunoprecipitation (ChIP) assays were performed in LS-8 cells. Lane 1 is a no template control using the test primers, lane 2 is the Pitx2 antibody immunoprecipitated (IP-Pitx2) chromatin, lane 3 is the normal rabbit immunoglobulin G (IgG) immunoprecipitated chromatin, and lane 4 is the input chromatin amplified using the test primers. The top gel (*Bmp4* enhancer) represents the test primers encompassing Pitx1/2 motif in the 396-bp *Bmp4* CRM. Lane 2 exhibits a 338 amplicon of the expected size. The lower two gels reveal the absence of amplicon products from the two control regions C1 and C2. An amplicon from these regions are only detected in the input control groups. (**B**) Quantitative real-time PCR was performed using the same conditions for the ChIP assays as described in (A). The 4.7-fold enrichment of the region containing the 396-bp *Bmp4* CRM in the Pitx2 antibody immunoprecipitated (Pitx2-IP) chromatin is shown in the bar graph. Significance is denoted by *p* = 0.0018 for the enrichment of amplified product in Pitx2-IP versus an IgG control set at 1.0. (**C**) Sequencing analysis demonstrates the presence of a Pitx1/2 binding site in the product amplified using the *Bmp4* minimal CRM region-specific primers and Pitx2-IP chromatin as template.

To determine whether Pitx2 physically binds the CONS3.8 *Bmp4* enhancer in living cells, we performed Chromatin Immunoprecipitation (ChIP) assays in LS8 mouse dental epithelial cells. LS8 cells were previously derived from the developing mouse enamel organ [51], and endogenously express *Pitx2* [50]. Using primers that target the Pitx1/2 binding motif in the 396 bp *Bmp4* enhancer sequence (CONS3.8), DNA purified from crosslinked LS8 chromatin after immunoprecipitation with an anti-Pitx2 antibody yielded a 4.7-fold increase in amplicon abundance, relative to an IgG control, by PCR and qPCR (Figure 6A, B). In contrast, the Pitx2-IP template did not support amplification for either of two control regions located 742 bp and 1807 bp upstream of CONS3.8, denoted C1 and C2 (Figure 6A). Furthermore, the specific amplicon was confirmed to contain the Pitx1/2 binding site by sequencing (Figure 6C). Thus, Pitx2 binds the conserved Pitx binding site in the *Bmp4* minimal enhancer *in vivo*.

## Discussion

### Pitx Homeoproteins as Potential Regulators of the *Bmp4* IE/LB Enhancer

We identified a 396 bp minimal ‘incisor epithelium/limb bud’ (IE/LB) *Bmp4* enhancer that contains putative binding sites for members of the Pitx and Msx homeoprotein families, which are expressed with *Bmp4* in these developing tissues. The Pitx and Msx binding sites reside 152 bp apart, and may represent part of a combinatorial code essential for *Bmp4* expression in early craniofacial and limb development. Both the Pitx and Msx binding motifs were present in all *Bmp4* IE/LB enhancer deletion constructs that exhibited reporter expression in transgenic assays, and mutation of either motif dramatically reduced expression. Interestingly, the entirety of the 396 bp *Bmp4* IE/LB enhancer, including the Pitx and Msx binding motifs, is also contained within the 467 bp lateral plate mesoderm (LPM) element described by Chandler and coworkers [1].

These data suggest specific possibilities about the regulatory circuitry that governs *Bmp4* expression in tooth and limb development. First, the presence of a required Pitx1/2 binding site in the *Bmp4* IE/LB enhancer supports a direct, positive regulatory relationship between Pitx1/2 and *Bmp4* gene expression. Interestingly, however, *Bmp4* expression is expanded in *Pitx2*-deficient mandibular ectoderm at E10.5, suggesting a repressive regulatory relationship [26], [52]. A similar expansion of *Bmp4* craniofacial expression is not observed in *Pitx1* null mutants [53], which exhibit defective mandibular development [48]. These data could be reconciled if, for example, Pitx1 and Pitx2, which are both expressed in initiation stage mandibular and incisor epithelium, functioned at slightly different times with a combination of activating and repressive capacities. Notably, *Pitx1* expression at the bud and cap stages of tooth development, including its exclusion from the enamel knot at the cap stage, mimics the persistent ectopic expression of the *Bmp4* IE/LB enhancer at these stages of tooth development (see Figure 1 and Figure S2, [19]). Conversely, at the cap stage, *Pitx2* expression is enriched in the enamel knot, from which *Bmp4* enhancer activity is absent. These data are consistent with a model whereby Pitx1 activates and Pitx2 represses the *Bmp4* enhancer in non-enamel knot dental epithelium and in enamel knot respectively, potentially through the same Pitx binding site in the *Bmp4* enhancer.

Although Pitx2 has been shown to function as a transcriptional activator, at least four known isoforms of Pitx2 exist via alternative splicing; these may have different regulatory properties. In addition, existing data indicates that Pitx2 can interact with any of several co-factors to regulate gene transcription [54]–[58]. It is therefore possible that co-factor choice may dictate whether the Pitx site in the *Bmp4* enhancer functions as a positive or negative regulatory input. For example, Pitx2 activation of the *Dlx2* promoter is attenuated by the direct interaction of Dlx2 with Pitx2 [58]. In another scenario involving the *Dlx2* promoter, Msx2 binds to the same Bicoid element as Pitx2, and antagonizes the activation of *Dlx2* transcription by Pitx2 [50]. These examples highlight the spectrum of possibilities that could account for the unique expression properties of the *Bmp4* IE/LB enhancer in developing incisor epithelium.

Thus, in early dental epithelium (mouse stage E9.5 - E11.5), Pitx1 or Pitx2 may activate the *Bmp4* enhancer directly by binding to the Pitx site. At later stages from E13.5, Bmp4 protein may establish a self-regulatory circuit, by negatively regulating *Pitx1* expression, as suggested by the observation of Bmp4-mediated *Pitx1* repression in dental epithelium [25], and by the transient repression of *Pitx2* by Bmp4 in mandibular epithelium [53]. Such a feedback loop could potentially explain the downregulation of *Bmp4* expression in the dental epithelium that normally occurs after the initiation stage. However, the Pitx site in the *Bmp4* enhancer itself is not a candidate to mediate this feedback since the *Bmp4* IE/LB enhancer fails to exhibit the normal downregulation observed for *Bmp4* expression in the incisor epithelium (Figure S2). Nonetheless, such a negative feedback loop might help ensure the correct regulation of Pitx1/2 mediated *Bmp4* expression. Indeed, *Pitx2* gene dosage is critical, because Axenfeld-Rieger patients carry *PITX2* haploinsufficiency, and *Pitx2+/−* mouse embryos exhibit oligodontia, along with other phenotypes [59].

Less is known about the relationship between *Bmp4* expression and Pitx1/2 in developing limb bud and lateral plate mesoderm, although similarly complex regulatory relationships may exist. *Pitx1* and *2* are differentially expressed in posterior and left sided lateral plate mesoderm, respectively, where *Pitx1* specifies hindlimb identity in LPM and *Pitx2* helps determine laterality [28]. Both *Pitx1* and *Pitx2* expression overlap in early LPM and they cooperate in hindlimb specification [28]. *Bmp4* expression is unaffected in the phenotypically affected hindlimbs of *Pitx1* null embryos [60], suggesting that other TFs, possibly Msx1/2 homeoproteins, regulate *Bmp4* expression in this tissue.

Finally, since mouse CONS3 has strong sequence homology with human, we performed bioinformatic analyses on the human sequence orthologous to mouse CONS3 to determine if it exhibits features of a regulatory enhancer. After aligning the mouse CONS3 to human sequence, we identified a 243-bp region of sequence homology located ∼45 kb upstream of the human *BMP4* gene. This region was compared against various epigenomic profiles in ENCODE human cell lines [61], [62]. Indeed, profiles of DNase I hypersensitivity, histone modification, and transcription factor binding all suggest that this region can function as an enhancer in human (Figure S7). Thus, public epigenomic data from ENCODE or other consortia can help corroborate potentially homologous enhancers in human.

In sum, one of the most interesting aspects of the present work is that a single enhancer can regulate the expression of a key developmental signaling gene such as *Bmp4* in craniofacial, limb and potentially lateral plate mesoderm tissues. This result furthers the view that while the fine details of gene regulatory networks may differ from case to case, the central features of these regulatory circuits are conserved, not only in evolution, but also in multiple developmental contexts within an organism.

## Materials and Methods

### Phylogenetic Footprinting (Comparative Genomic Analysis of Conserved Sequences)

To generate human, mouse and pufferfish alignments, mouse genomic sequence surrounding the *Bmp4* gene (76 kb upstream and 76 kb downstream of the *Bmp4* gene) from public database (GenBank accession X56848.1) was compared against orthologous human and pufferfish sequences using BLASTZ (http://bio.cse.psu.edu/pipmaker/), a local alignment program that generates graphical outputs by PipMaker as blocks of sequence conservation [34], [35]. Repetitive DNA of the reference mouse sequence was masked using RepeatMasker (http://www.repeatmasker.org/cgi-bin/WEBRepeatMasker).

### Generation of DNA Constructs for Microinjection

The blocks of conserved non-coding sequences (CONS regions) were amplified by polymerase chain reaction (PCR) using either MasterAmp PCR amplification or Extralong PCR amplification kit with proofreading DNA polymerase (Epicentre Biotechnologies, Madison, WI). The PCR primers were designed to contain a restriction site and an additional 3–6 nucleotides at their 5′ ends for subsequent restriction enzyme digestion and directional cloning of the PCR product. All constructs CONS3.1, 3.2, 3.3, 3.4, 3.5 were subcloned directly from CONS3 construct using restriction enzyme digestion.

The PCR products were purified using QIAquick PCR Purification Kit (QIAGEN, Valencia, CA), restriction enzyme-digested and subcloned into a multiple cloning site of GLKS plasmid which contains an *E. coli lacZSV40pA* reporter cassette under the control of a minimal human β-globin promoter [63]. Correct clones were confirmed by either restriction enzyme digestion or direct sequencing methods. Constructs were released from GLKS vector backbone using appropriate restriction enzymes, purified using QIAGEN Gel Extraction kit (QIAGEN, Valencia, CA) and eluted with microinjection buffer (10 mM Tris pH 8.0, 0.25 mM EDTA).

### Generation and Genotyping of Transgenic Mice

The constructs were diluted in microinjection buffer to a concentration of 1–2 ng/µl and injected into fertilized mouse oocytes derived from FVB/N matings using standard procedures [64]. The injected oocytes were then transferred into oviducts of pseudopregnant Swiss female mice. Transgenic embryos were collected, fixed and stained for β-galactosidase activity. Yolk sacs were carefully dissected away from maternal tissues and avoided cross contaminations between littermates. Crude yolk sac genomic DNA was extracted by overnight digestion in 200 µl of yolk sac lysis buffer (50mM potassium chloride, 1.5mM magnesium chloride, 10mM Tris pH8.3, 0.01% gelatin, 0.45% Nonidet P-40, 0.45% Tween-20) supplemented with 0.1mg/ml proteinase K at 55°C. For tail biopsies, genomic DNA was extracted by boiling the tissue for 20 minutes in 200 µl of 25mM Sodium hydroxide, followed by neutralization with 50 µl of Tris-HCl pH 8.0. Transgenic embryos and transgenic founder mice were identified by the presence of the *lacZ* transgene using PCR with primers wlacZ-F 5′-TTCACTGGCCGTCGTTTTACAACGTCG-3′ and wlacZ-R 5′-ATGTGAGCGAGTAACAACCCGTCGGA-3′. Permanent transgenic lines were established by crossing the founder animal with FVB/N wild type mice. Age-matched *Bmp4^lacZneo^* knockin heterozygous embryos were used as controls. The control embryos were derived from crossing *Bmp4^lacZneo^* heterozygous male mice with wild type CD-1 or ICR female mice. The animal use protocol was reviewed and approved by the Institutional Animal Care and Use Committee (IACUC) of Harvard Medical School.

### Cryosections and Whole Mount β-galactosidase Staining

Staining for β-galactosidase activity in whole embryos and on cryosections was performed according to standard protocols [64]. Heads of selected transgenic embryos were removed and the lower jaws were separated to allow direct visualization of the intraoral cavity and to facilitate penetration of X-gal staining solution. For cryosections, embryonic heads were processed as described and sectioned at 10 µm thickness. After staining for β-galactosidase activity, the sections were counterstained with 0.5% Eosin Y solution, dehydrated through an ascending series of ethyl alcohol, cleared in xylene and mounted with Permount (Sigma, St. Louis, MO).

### Laser Capture Microdissection (LCM) and Gene Expression Analysis

Embryonic tissue was dissected in ice-cold RNase-free phosphate buffered saline (PBS) and the E11.0 heads were frozen immediately in Tissue Tek OCT (Andwin Scientific, Schaumburg, IL). Fresh-frozen tissue was cryosectioned and collected on PEN membrane slides (Molecular Devices, Sunnyvale, CA). The slides were immediately refrozen and maintained on dry ice before staining and dehydrating with Histogene Staining Kit (Molecular Devices, Sunnyvale, CA). Discrete incisor epithelial tissues were isolated using a Leica laser capture microdissection (LCM) LMD 6000 microscope. The tissue was isolated directly into the extraction buffer provided with Pico Pure Isolation Kit (Molecular Devices, Sunnyvale, CA). RNA purification was performed according to the Pico Pure Isolation Kit and included an on column treatment with RNase-free DNase I (Qiagen, Valencia, CA). Eluted RNA quality was determined using the Agilent Bioanalyzer 2100. 10 to 25 ng of total RNA was then amplified to yield 7–10 µg of single-stranded DNA using a poly-dT based Ovation RNA Amplification System V2 (NuGEN, San Carlos, CA). The quality and size distribution of amplified DNA were confirmed using an Agilent Bioanalyzer 2100. Biotinylation was achieved through abasic site creation in the single stranded DNA with Uracil N-Glycosylase (Epicentre Biotechnologies, Madison, WI), and reaction with Aldehyde-Reactive Probe (ARP) N-(aminooxyacetyl)-N′-(D-biotinoyl) hydrazine, trifluoroacetic acid salt (Invitrogen). 1.5 µg of biotinylated DNA was hybridized according to the NuGEN manufacture note to the Illumina Mouse Ref-6 whole genome expression array. For expression profiling of the developing limb, Affymetrix microarray datasets were obtained from Gene Expression Omnibus (GEO) reference series GSE2560. Probe datasets were called “Present” if at least two of the three replicates had a detection *p*-value less than 0.05. The E11.0 tooth germ microarray data is deposited in the GEO database (http://www.ncbi.nih.gov/geo/).

### Analysis of the CONS3.8 CRM for Putative TF binding sites

Custom MATLAB scripts were written to map ungapped 8-mers from the UniPROBE database for mouse TFs ([20], http://thebrain.bwh.harvard.edu/uniprobe/) to the CONS3.8 CRM. The Mouse July 2007 (NCBI37/mm9) assembly was used along with the 46-way multiz vertebrate alignment from Galaxy (http://main.g2.bx.psu.edu/). A putative binding site in CONS3.8 was selected for further analysis when at least two consecutive overlapping ungapped 8-mers scored above an enrichment score (ES) of 0.35 (medium affinity binding site) and 0.45 (high affinity binding site). We focused on TF families whose members had binding sites that were conserved in at least 90% of the vertebrate alignment within CONS3.8. TFs were grouped into families based on their names and their common DNA binding domain (DBD) annotation from the InterPro database [65]; (*e.g.,* since the TFs Msx1, Msx2, and Msx3 each have the same name, Msx, and the same three DBD annotations of IPR000047 (helix-turn-helix), IPR001356 (homeodomain), and IPR009057 (homeodomain-like), we grouped these three TFs into the family “Msx IPR000047; IPR001356; IPR009057”). We further restricted our candidate TF families based on the expression of at least one of their members in the tissues of interest. Microarray datasets were used from mouse tissue of E11.5 tooth germ (this study) and E12.5 fore- (GEO datasets GSM48648, GSM48912, GSM48913) and hindlimb (GEO datasets GSM48914, GSM48915, GSM48916). Transcription factors were retained if they were called present in at least 2 out of the 3 replicates, in all three tissues, and had conserved binding sites in CONS3.8.

### Generation of Pitx1 and Msx2 Plasmid Constructs

Full-length Pitx1 and Msx2 expression plasmids were constructed in pGEX3X by PCR-based cloning of the Pitx1 and Msx2 coding regions. Each reaction contained template, 4 pmoles of each primer, 2.5 U of PfuTurbo® DNA polymerase (Stratagene, La Jolla, CA), and 2× FailSafe PreMix A (Epicentre Biotechnologies, Madison, WI) which contains 1.5 mM Mg^2+^and 1× Betaine. PCR conditions were as follows: 95°C for 2 minutes, 95°C for 30 seconds, 60°C for 30 seconds, and 72°C for 1 minute with a final extension of 10 minutes after 30 cycles in PCR thermocyclers (MJ Research, Reno, NV). We purified the PCR products using Gel Extraction kit (QIAGEN, Valencia, CA). Approximately 1 µg of the purified PCR products was subjected to double digestion. The digested products were then ligated into the expression vector (pGEX3X). Both Pitx1 and Msx2 constructs were directly sequenced with forward and reverse primers designed from plasmid sequences flanking the cloning site (pGEX3X-F: 5′-ATGGCCTTTGCAGGGCTGGCAAGC-3′ and pGEX3X-R: 5′-TCTCCGGGAGCTGCAT-GTGTCAG-3′) to ensure the absence of mutations.

### Pitx1 and Msx2 Glutathione S-Transferase Fusion Protein Preparation

Cells of *Escherichia coli* strain BL21 (DE3) was transformed using the clones selected after confirming the sequences. Bacterial cultures were induced with 1.0 mM isopropyl-1-thio-ß-D-galactopyranoside (IPTG) for 3 hours. Cells were collected after centrifugation at 5000x*g* for 30 minutes at 4°C. The cultures were resuspended in ice-cold phosphate buffered saline (PBS). Cell lysis was performed by adding lysozyme to a final concentration of 1 mg/ml and incubation on ice for 30 minutes. Triton X-100, DNase I and 5 µg/µl RNase A and 1 mM PMSF were added to the lysates. After incubation at 4°C for 30 minutes, lysates were spun at 3000xg for 30 minutes. Supernatants were removed and adjusted to 1 mM DTT and 1 mM PMSF. Fusion proteins were purified using glutathione agarose beads (Sigma, St. Louise, MO) according to standard protocols.

### Preparation of Double Stranded DNA Targets

Two control target DNA sequences used for Pitx1 and Msx2 EMSA were Bicoid: 5′-TCATGCCTGTAATCCCAGCACTCAG-3′ and MBS: 5′-GATCCACTAATTGGAGG-3′, respectively [49], [50]. Target DNAs for Pitx1-CONS3 EMSA and Msx2-CONS3 EMSA were WTPitxBSCONS3∶5′-AGTTCCCTACATAATCCTTACCGTG-3′, MutPitxBSCONS3∶5′-AGTTCCCTACAACGCATTTACCGTG-3′, WTMsxBSCONS3∶5′-GACCCTATGTA-ATTGCATTCCTGAA-3′and MutMsxBSCONS3∶5′-GACCCTATCCGGCCTCATTCCTGAA-3′. Four micrograms of each of the above synthetic oligonucleotide pairs were annealed by boiling for 5 min. in annealing buffer (10 mM Tris, pH 7.5, 50 mM NaCl, 1mM MgCl_2_) followed by cooling. They were then labeled in a 20 µl reaction volume using 20 ng of annealed oligonucleotide in polynucleotide kinase (PNK) buffer (New England BioLabs, Inc., Beverly, MA), 10 units of T4 polynucleotide kinase (New England BioLabs, Beverly, MA) and 5 µCi of γ-^32^P-ATP. Reactions were incubated at 37°C for 1 hour. To remove unincorporated oligonucleotides, the labeled probe reactions were loaded onto Micro Bio-Spin P-30 Tris Chromatography columns (Bio-RAD, Hercules, CA) and centrifuged according to the manufacturer’s instructions and diluted with protein binding buffer to ∼2×10^4^ cpm/µg.

### Electrophoretic Mobility Shift Assay (EMSA)

Protein-DNA binding reactions of 20 µl total volume were performed by adding increasing amounts of purified fusion proteins in final reaction of 1X phosphate buffer, 10% glycerol, 0.3 mg/µl BSA and 0.1 µg/µl DNaseI digested poly[dG-dC], incubated on ice for 10 minutes and then further incubated with addition of 1 µl of [^32^P]-labeled annealed probe on ice for 20 minutes. One microliter of loading dye was added to a free probe reaction (no protein) to assist in locating free probe in the gel. Protein-DNA complexes were separated on a pre-cooled 6% non-denaturing polyacrylamide gel. Electrophoresis was performed at 4°C with 1x Tris-Borate-EDTA (TBE) buffer at 200 volts for 2 hours. Gels were transferred to Whatman paper and dried at 80°C for 2 hours before being subjected to autoradiography at –80°C.

### *In vitro* Mutagenesis

We used a QuikChange Lightning Site-Directed Mutagenesis Kit (Clontech, Mountainview, CA) and the following primer pairs to perform mutagenesis of Pitx or Msx binding sites in the CONS3 construct - SDMPitxBS-F: 5′-GAGGGCTCTTCACGGTAAatgcgtTGTAGGGAA CTTAAAAAGAAG-3′, SDMPitxBS-R: 5′-CTTCTTTTTAAGTTCCCTACAacgcatTTACCG TGAAGAGCCCTC-3′ and SDMMsxBS-F: 5′-GGGCCTGTTACTCCTTCAGGAATG aggccggATAGGGTCAAATA-AAACATG-3′, SDMMsxBS-R: 5′-CATGTTTTATTTGACCC TATccggcctCATTCCTGAAG-GAGTAACAGGCCC-3′. The mutated constructs were sequenced to confirm the introduced mutation prior to purification for pronuclear microinjection. E11.5 transgenic embryos were collected and tested for reporter activity as described above.

### Chromatin Immunoprecipitation (ChIP) Assay

The ChIP assays were performed as previously described [56] using the ChIP Assay Kit (Upstate/Millipore, Billerica, MA) with the following modifications. LS-8 cells were fed for 24 hours, harvested and plated in 60 mm dishes, then cross-linked with 1% formaldehyde for 10 minutes at 37°C the next day. All PCR reactions were done under an annealing temperature of 61°C. The primers for amplifying the Pitx2 binding site in the *Bmp4* CONS3 are as follows: forward 5′-CCACCCACAGATTCAGACCT-3′ and reverse 5′-CAGGAAGGAAT- TCGAAGCAG-3′ (chr14∶47,056,511–47,056,848). The two control primers are as follows: forward 5′-AGCAAACAGGCGATCTCATT-3′ and reverse 5′- GGAGTGGTGAA- GGTCTTGGA-3′ (chr14∶47,057,304–47,057,590); forward 5′-TGCATGTGGTCAGTCAGTCA-3′ and reverse 5′-TGCTTCACCACAGGTCTCAG-3′ (chr14∶47,058,298–47,058,655). All the PCR products were evaluated on a 2% agarose gel in 1X TBE for appropriate size. Quantitative real-time PCR was performed using the same annealing temperature but extending the number of cycles. Identical amounts of the IP DNA and IgG DNA were loaded as template. All of the regular PCRs and real-time PCR products were confirmed by sequencing.

## Supporting Information

Figure S1 ***Bmp4^lacZneo^*** **and** ***Tg^CONS3^*** **β-gal activity in initiation stage incisor.** Whole mount and sagittal sections of *Bmp4^lacZneo^* and *Tg^CONS3^* transgenic upper incisors. Abbr: NP, nasal pit; MNP, medial nasal process; MxP, maxillary process; MdP, mandibular process; OC, oral cavity; UI, upper incisor; M, mesenchyme; DL, dental lamina; D, dorsal; V, ventral; A, anterior; P, posterior.
(TIF)

Figure S2 ***Bmp4^lacZneo^*** **and** ***Tg^CONS3.5^*** **β-gal activity in cap-stage incisors.** Sagittal sections of cap stage *Bmp4^lacZneo^* and *Tg^CONS3^.^5^* transgenic upper and lower incisors. Note persistent inappropriate expression of *Tg^CONS3.5^* in non-enamel knot dental epithelium and surrounding ectoderm, and its failure to be expressed in the dental papilla and adjacent mesenchyme, as is observed in the control *Bmp4^lacZneo^* heterozygote. Abbr: EK, enamel knot; DP, dental papilla; EO, enamel organ; T, tongue; UL, upper lip; LL, lower lip; VL, vestibular lamina.
(TIF)

Figure S3 **Laser capture microdissection (LCM) of epithelial incisor region.** Sagittal sections of an E11.5 initiation stage incisor pre- and post-LCM. The captured epithelium was identified by bright field microscopy. Scale bar: 100 µm.
(TIF)

Figure S4 **Pitx and Msx binding sites in the** ***Bmp4*** **IE/LB enhancer.** Sequence of the 758 bp region (chr14∶47,056,171–47,056,928) containing the 396 bp *Bmp4* minimal enhancer double underlined (chr14∶47,056,283–47,056,678). The Pitx1/2 binding site (5′-TAATCC-3′) and Msx1/2 binding site (5′-GTAATTG-3′) are indicated, and the location of the 467 bp enhancer (chr14∶47,056,226–47,056,692) described by Chandler and coworkers [1] containing the 396 bp minimal enhancer sequence contained is underlined.
(TIF)

Figure S5 ***In vitro*** **binding of Msx to the minimal** ***Bmp4*** **enhancer.** Electrophoretic Mobility Shift Assay (EMSA) exhibits robust binding of Msx2 protein to both a positive control MBS DNA sequence and to the consensus Msx1/2 binding site in the CONS3 sequence. Competition with specific or non-specific cold probes indicates sequence-specific binding of GST-Msx2 fusion protein to the consensus Msx1/2-binding motif.
(TIF)

Figure S6 **Transgenic mutational analysis of Pitx and Msx binding sites.** Graphical representation of the transgenic mutational results presented in Fig. 5B indicates that Pitx binding site is necessary for reporter expression in forelimb and hindlimb AER and mesenchyme and orofacial tissues.
(TIF)

Figure S7 **Integrative epigenomic analysis suggests that the human orthologous sequence of mouse CONS3 likely functions as an enhancer.** Using public epigenomic data from the University of California at Santa Cruz (UCSC) Genome Browser, we analyzed the human homolog of CONS3 (labeled CONS3 at the upper right hand corner of the top genome browser view; genome assembly hg18). This region is enriched for an enhancer associated histone mark H3K4me1, is DNaseI hypersensitive, is a binding site for transcription factors Max and c-Fox in human embryonic stem cells, and is annotated to be in “enhancer state” in multiple human cell lines by ChromHMM [62]. These multiple lines of evidence suggest CONS3 homolog may also function as an enhancer in human.
(TIF)

Table S1 **Families of Transcription Factors (TFs) with their InterPro (IPR) DNA binding domain annotation number that have potential binding sequences in the minimal** ***Bmp4*** **enhancer.** Transcription factors families with binding sites (BS) in the minimal *Bmp4* enhancer in >75% and >90% of aligned vertebrates are indicated.
(PDF)

## References

[1] Chandler KJ, Chandler RL, Mortlock DP (2009). Identification of an ancient Bmp4 mesoderm enhancer located 46 kb from the promoter. Dev Biol 327: 590–602.. doi:10.1016/j.ydbio.2008.12.033.

[2] Hogan BL (1996). Bone morphogenetic proteins in development.. Curr Opin Genet Dev.

[3] Zhao G-Q (2003). Consequences of knocking out BMP signaling in the mouse. Genesis 35: 43–56.. doi:10.1002/gene.10167.

[4] Jones CM, Lyons KM, Hogan BL (1991). Involvement of Bone Morphogenetic Protein-4 (BMP-4) and Vgr-1 in morphogenesis and neurogenesis in the mouse.. Development.

[5] Hogan BL (1996). Bone morphogenetic proteins: multifunctional regulators of vertebrate development.. Genes Dev.

[6] Andl T, Ahn K, Kairo A, Chu EY, Wine-Lee L (2004). Epithelial Bmpr1a regulates differentiation and proliferation in postnatal hair follicles and is essential for tooth development. Development 131: 2257–2268.. doi:10.1242/dev.01125.

[7] Liu W, Selever J, Murali D, Sun X, Brugger SM (2005). Threshold-specific requirements for Bmp4 in mandibular development. Dev Biol 283: 282–293.. doi:10.1016/j.ydbio.2005.04.019.

[8] Robert B (2007). Bone morphogenetic protein signaling in limb outgrowth and patterning. Dev Growth Differ 49: 455–468.. doi.

[9] Selever J, Liu W, Lu M-F, Behringer RR, Martin JF (2004). Bmp4 in limb bud mesoderm regulates digit pattern by controlling AER development. Dev Biol 276: 268–279.. doi:10.1016/j.ydbio.2004.08.024.

[10] Liu W, Sun X, Braut A, Mishina Y, Behringer RR (2005). Distinct functions for Bmp signaling in lip and palate fusion in mice. Development 132: 1453–1461.. doi:10.1242/dev.01676.

[11] Chen Y, Bei M, Woo I, Satokata I, Maas R (1996). Msx1 controls inductive signaling in mammalian tooth morphogenesis.. Development.

[12] Ohazama A, Tucker A, Sharpe PT (2005). Organized tooth-specific cellular differentiation stimulated by BMP4.. J Dent Res.

[13] Plikus MV, Zeichner-David M, Mayer J-A, Reyna J, Bringas P (2005). Morphoregulation of teeth: modulating the number, size, shape and differentiation by tuning Bmp activity. Evol Dev 7: 440–457.. doi.

[14] Ahn K, Mishina Y, Hanks MC, Behringer RR, Crenshaw EB 3rd (2001). BMPR-IA signaling is required for the formation of the apical ectodermal ridge and dorsal-ventral patterning of the limb.. Development.

[15] Pizette S, Abate-Shen C, Niswander L (2001). BMP controls proximodistal outgrowth, via induction of the apical ectodermal ridge, and dorsoventral patterning in the vertebrate limb.. Development.

[16] Wang C-KL, Omi M, Ferrari D, Cheng H-C, Lizarraga G (2004). Function of BMPs in the apical ectoderm of the developing mouse limb. Dev Biol 269: 109–122.. doi:10.1016/j.ydbio.2004.01.016.

[17] Bandyopadhyay A, Tsuji K, Cox K, Harfe BD, Rosen V (2006). Genetic analysis of the roles of BMP2, BMP4, and BMP7 in limb patterning and skeletogenesis. PLoS Genet 2: e216.. doi:10.1371/journal.pgen.0020216.

[18] Bénazet J-D, Bischofberger M, Tiecke E, Gonçalves A, Martin JF (2009). A self-regulatory system of interlinked signaling feedback loops controls mouse limb patterning. Science 323: 1050–1053.. doi:10.1126/science.1168755.

[19] O’Connell DJ, Ho JWK, Mammoto T, Turbe-Doan A, O’Connell JT (2012). A Wnt-bmp feedback circuit controls intertissue signaling dynamics in tooth organogenesis. Sci Signal 5: ra4.. doi:10.1126/scisignal.2002414.

[20] Newburger DE, Bulyk ML (2009). UniPROBE: an online database of protein binding microarray data on protein-DNA interactions. Nucleic Acids Res 37: D77–82.. doi:10.1093/nar/gkn660.

[21] Robasky K, Bulyk ML (2011). UniPROBE, update 2011: expanded content and search tools in the online database of protein-binding microarray data on protein-DNA interactions. Nucleic Acids Res 39: D124–128.. doi:10.1093/nar/gkq992.

[22] Semina EV, Reiter R, Leysens NJ, Alward WL, Small KW (1996). Cloning and characterization of a novel bicoid-related homeobox transcription factor gene, RIEG, involved in Rieger syndrome. Nat Genet 14: 392-399.. doi.

[23] Lanctôt C, Lamolet B, Drouin J (1997). The bicoid-related homeoprotein Ptx1 defines the most anterior domain of the embryo and differentiates posterior from anterior lateral mesoderm.. Development.

[24] Gurnett CA, Alaee F, Kruse LM, Desruisseau DM, Hecht JT (2008). Asymmetric lower-limb malformations in individuals with homeobox PITX1 gene mutation. Am J Hum Genet 83: 616–622.. doi:10.1016/j.ajhg.2008.10.004.

[25] Mitsiadis TA, Drouin J (2008). Deletion of the Pitx1 genomic locus affects mandibular tooth morphogenesis and expression of the Barx1 and Tbx1 genes. Dev Biol 313: 887–896.. doi:10.1016/j.ydbio.2007.10.055.

[26] Lin CR, Kioussi C, O’Connell S, Briata P, Szeto D (1999). Pitx2 regulates lung asymmetry, cardiac positioning and pituitary and tooth morphogenesis. Nature 401: 279–282.. doi:10.1038/45803.

[27] Lu MF, Pressman C, Dyer R, Johnson RL, Martin JF (1999). Function of Rieger syndrome gene in left-right asymmetry and craniofacial development. Nature 401: 276–278.. doi:10.1038/45797.

[28] Marcil A, Dumontier E, Chamberland M, Camper SA, Drouin J (2003). Pitx1 and Pitx2 are required for development of hindlimb buds.. Development.

[29] Thomas JW, Touchman JW, Blakesley RW, Bouffard GG, Beckstrom-Sternberg SM (2003). Comparative analyses of multi-species sequences from targeted genomic regions. Nature 424: 788–793.. doi:10.1038/nature01858.

[30] Lenhard B, Sandelin A, Mendoza L, Engström P, Jareborg N (2003). Identification of conserved regulatory elements by comparative genome analysis. J Biol 2: 13.. doi.

[31] Wasserman WW, Sandelin A (2004). Applied bioinformatics for the identification of regulatory elements. Nat Rev Genet 5: 276–287.. doi:10.1038/nrg1315.

[32] Feng JQ, Chen D, Cooney AJ, Tsai MJ, Harris MA (1995). The mouse bone morphogenetic protein-4 gene. Analysis of promoter utilization in fetal rat calvarial osteoblasts and regulation by COUP-TFI orphan receptor.. J Biol Chem.

[33] Kurihara T, Kitamura K, Takaoka K, Nakazato H (1993). Murine bone morphogenetic protein-4 gene: existence of multiple promoters and exons for the 5′-untranslated region. Biochem Biophys Res Commun 192: 1049–1056.. doi:10.1006/bbrc.1993.1523.

[34] Schwartz S, Zhang Z, Frazer KA, Smit A, Riemer C (2000). PipMaker–a web server for aligning two genomic DNA sequences.. Genome Res.

[35] Schwartz S, Kent WJ, Smit A, Zhang Z, Baertsch R (2003). Human-mouse alignments with BLASTZ. Genome Res 13: 103–107.. doi:10.1101/gr.809403.

[36] Ahituv N, Rubin EM, Nobrega MA (2004). Exploiting human–fish genome comparisons for deciphering gene regulation. Hum Mol Genet 13 Spec No 2: R261–266.. doi:10.1093/hmg/ddh229.

[37] Koop BF, Nadeau JH (1996). Pufferfish and new paradigm for comparative genome analysis.. Proc Natl Acad Sci USA.

[38] Nobrega MA, Ovcharenko I, Afzal V, Rubin EM (2003). Scanning human gene deserts for long-range enhancers. Science 302: 413.. doi:10.1126/science.1088328.

[39] Loots GG, Locksley RM, Blankespoor CM, Wang ZE, Miller W (2000). Identification of a coordinate regulator of interleukins 4, 13, and 5 by cross-species sequence comparisons.. Science.

[40] Santagati F, Abe K, Schmidt V, Schmitt-John T, Suzuki M (2003). Identification of Cis-regulatory elements in the mouse Pax9/Nkx2-9 genomic region: implication for evolutionary conserved synteny.. Genetics.

[41] Bejerano G, Haussler D, Blanchette M (2004). Into the heart of darkness: large-scale clustering of human non-coding DNA. Bioinformatics 20 Suppl 1: i40–48.. doi:10.1093/bioinformatics/bth946.

[42] Van den Wijngaard A, Pijpers MA, Joosten PH, Roelofs JM, Van zoelenE J (1999). Functional characterization of two promoters in the human bone morphogenetic protein-4 gene. J Bone Miner Res 14: 1432–1441.. doi:10.1359/jbmr.1999.14.8.1432.

[43] Feng JQ, Zhang J, Tan X, Lu Y, Guo D (2002). Identification of cis-DNA regions controlling Bmp4 expression during tooth morphogenesis in vivo.. J Dent Res.

[44] Zhang J, Tan X, Contag CH, Lu Y, Guo D (2002). Dissection of promoter control modules that direct Bmp4 expression in the epithelium-derived components of hair follicles. Biochem Biophys Res Commun 293: 1412–1419.. doi.

[45] Lawson KA, Dunn NR, Roelen BA, Zeinstra LM, Davis AM (1999). Bmp4 is required for the generation of primordial germ cells in the mouse embryo.. Genes Dev.

[46] Aberg T, Wozney J, Thesleff I (1997). AID-AJA3>3.0.CO;2-C..

[47] Thesleff I, Keränen S, Jernvall J (2001). Enamel knots as signaling centers linking tooth morphogenesis and odontoblast differentiation.. Adv Dent Res.

[48] Lanctôt C, Moreau A, Chamberland M, Tremblay ML, Drouin J (1999). Hindlimb patterning and mandible development require the Ptx1 gene.. Development.

[49] Semenza GL, Wang GL, Kundu R (1995). DNA binding and transcriptional properties of wild-type and mutant forms of the homeodomain protein Msx2. Biochem Biophys Res Commun 209: 257–262.. doi:10.1006/bbrc.1995.1497.

[50] Green PD, Hjalt TA, Kirk DE, Sutherland LB, Thomas BL (2001). Antagonistic regulation of Dlx2 expression by PITX2 and Msx2: implications for tooth development.. Gene Expr.

[51] Zhou YL, Snead ML (2000). Identification of CCAAT/enhancer-binding protein alpha as a transactivator of the mouse amelogenin gene.. J Biol Chem.

[52] Liu W, Selever J, Lu M-F, Martin JF (2003). Genetic dissection of Pitx2 in craniofacial development uncovers new functions in branchial arch morphogenesis, late aspects of tooth morphogenesis and cell migration. Development 130: 6375–6385.. doi:10.1242/dev.00849.

[53] St Amand TR, Zhang Y, Semina EV, Zhao X, Hu Y (2000). Antagonistic signals between BMP4 and FGF8 define the expression of Pitx1 and Pitx2 in mouse tooth-forming anlage. Dev Biol 217: 323–332.. doi:10.1006/dbio.1999.9547.

[54] Poulin G, Lebel M, Chamberland M, Paradis FW, Drouin J (2000). Specific protein-protein interaction between basic helix-loop-helix transcription factors and homeoproteins of the Pitx family.. Mol Cell Biol.

[55] Tremblay JJ, Goodyer CG, Drouin J (2000). Transcriptional properties of Ptx1 and Ptx2 isoforms.. Neuroendocrinology.

[56] Amen M, Liu X, Vadlamudi U, Elizondo G, Diamond E (2007). PITX2 and beta-catenin interactions regulate Lef-1 isoform expression. Mol Cell Biol 27: 7560–7573.. doi.

[57] Cao H, Florez S, Amen M, Huynh T, Skobe Z (2010). Tbx1 regulates progenitor cell proliferation in the dental epithelium by modulating Pitx2 activation of p21. Dev Biol 347: 289–300.. doi:10.1016/j.ydbio.2010.08.031.

[58] Venugopalan SR, Li X, Amen MA, Florez S, Gutierrez D (2011). Hierarchical interactions of homeodomain and forkhead transcription factors in regulating odontogenic gene expression. J Biol Chem 286: 21372–21383.. doi:10.1074/jbc.M111.252031.

[59] Gage PJ, Suh H, Camper SA (1999). Dosage requirement of Pitx2 for development of multiple organs.. Development.

[60] Szeto DP, Rodriguez-Esteban C, Ryan AK, O’Connell SM, Liu F (1999). Role of the Bicoid-related homeodomain factor Pitx1 in specifying hindlimb morphogenesis and pituitary development.. Genes Dev.

[61] Myers RM, Stamatoyannopoulos J, Snyder M, Dunham I, Hardison RC (2011). A user’s guide to the encyclopedia of DNA elements (ENCODE). PLoS Biol 9: e1001046.. doi:10.1371/journal.pbio.1001046.

[62] Ernst J, Kheradpour P, Mikkelsen TS, Shoresh N, Ward LD (2011). Mapping and analysis of chromatin state dynamics in nine human cell types. Nature 473: 43–49.. doi:10.1038/nature09906.

[63] Zhang X, Friedman A, Heaney S, Purcell P, Maas RL (2002). Meis homeoproteins directly regulate Pax6 during vertebrate lens morphogenesis. Genes Dev 16: 2097–2107.. doi:10.1101/gad.1007602.

[64] Nagy A (2003). Manipulating the mouse embryo: a laboratory manual. CSHL Press.. 776 p.

[65] Hunter S, Jones P, Mitchell A, Apweiler R, Attwood TK (2012). InterPro in 2011: new developments in the family and domain prediction database. Nucleic Acids Res 40: D306–312. doi:10.1093/nar/gkr948. InterPro Website.. http://www.ebi.ac.uk/interpro/(Accessed.

