# Supplementary figures and images for: An Evolutionarily Conserved Enhancer Regulates Bmp4 Expression in Developing Incisor and Limb Bud
Source: PLoS One. 2012 Jun 12;7(6):e38568. doi: 10.1371/journal.pone.0038568 (PMC3373496; doi:10.1371/journal.pone.0038568)

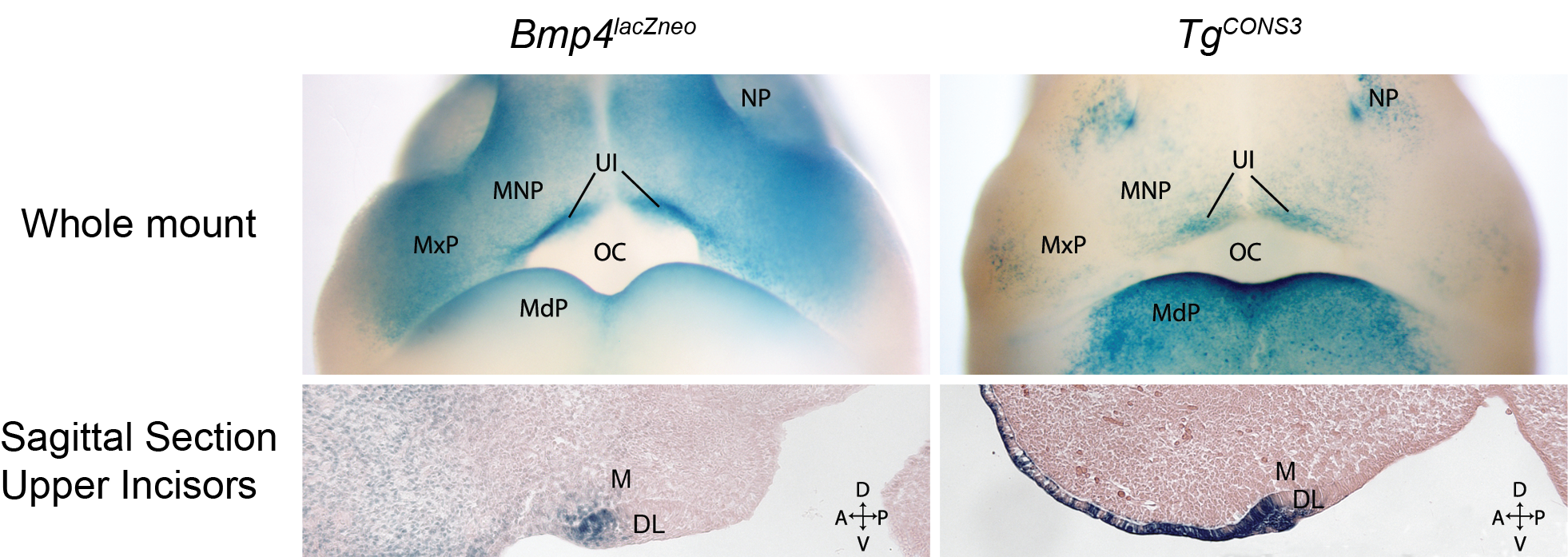

Supplement: Figure S1 — Bmp4lacZneo and TgCONS3 β-gal activity in initiation stage incisor. Whole mount and sagittal sections of Bmp4lacZneo and TgCONS3 transgenic upper incisors. Abbr: NP, nasal pit; MNP, medial nasal process; MxP, maxillary process; MdP, mandibular process; OC, oral cavity; UI, upper incisor; M, mesenchyme; DL, dental lamina; D, dorsal; V, ventral; A, anterior; P, posterior. (TIF) [file pone.0038568.s001.tif]

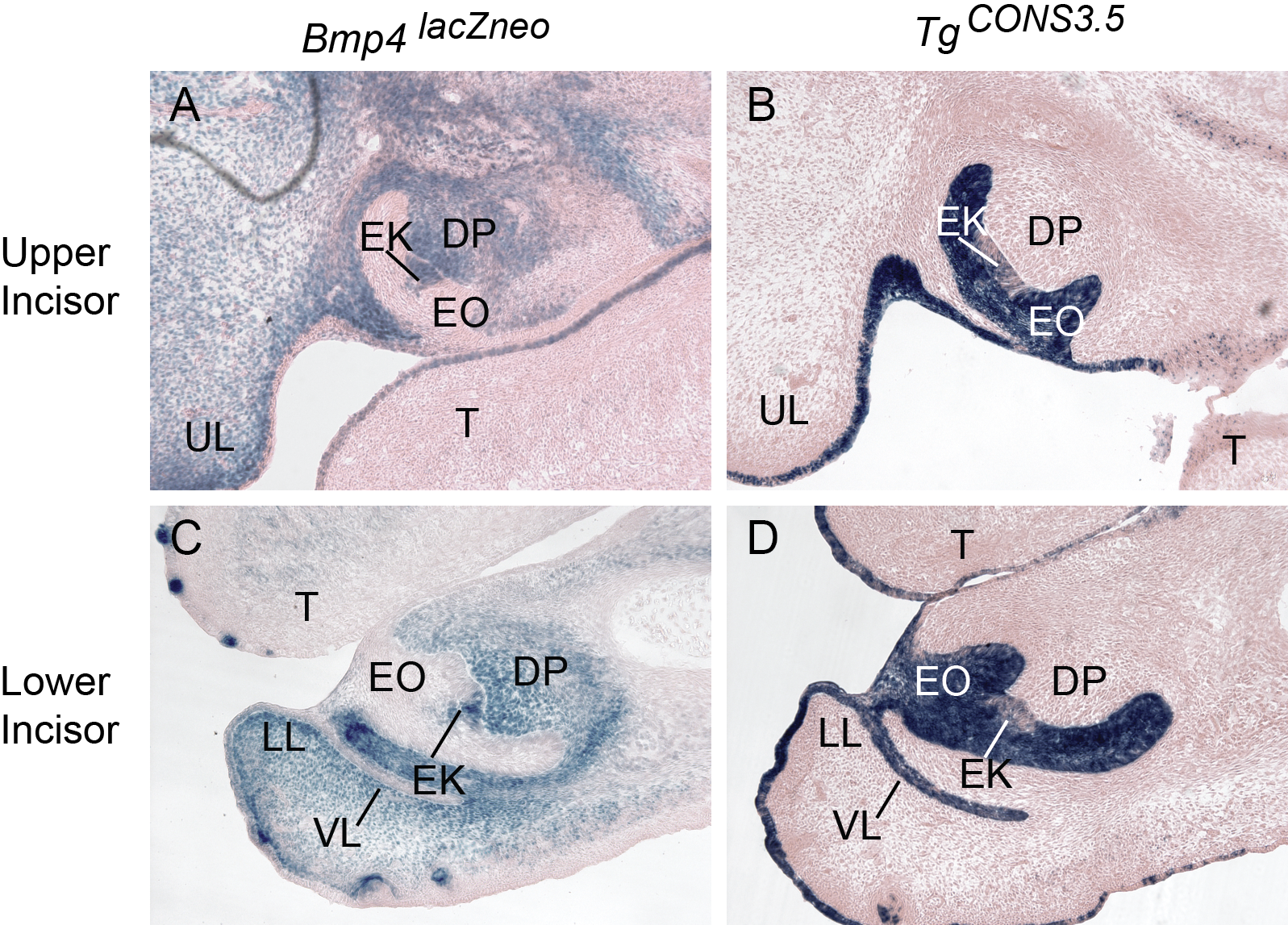

Supplement: Figure S2 — Bmp4lacZneo and TgCONS3.5 β-gal activity in cap-stage incisors. Sagittal sections of cap stage Bmp4lacZneo and TgCONS3.5 transgenic upper and lower incisors. Note persistent inappropriate expression of TgCONS3.5 in non-enamel knot dental epithelium and surrounding ectoderm, and its failure to be expressed in the dental papilla and adjacent mesenchyme, as is observed in the control Bmp4lacZneo heterozygote. Abbr: EK, enamel knot; DP, dental papilla; EO, enamel organ; T, tongue; UL, upper lip; LL, lower lip; VL, vestibular lamina. (TIF) [file pone.0038568.s002.tif]

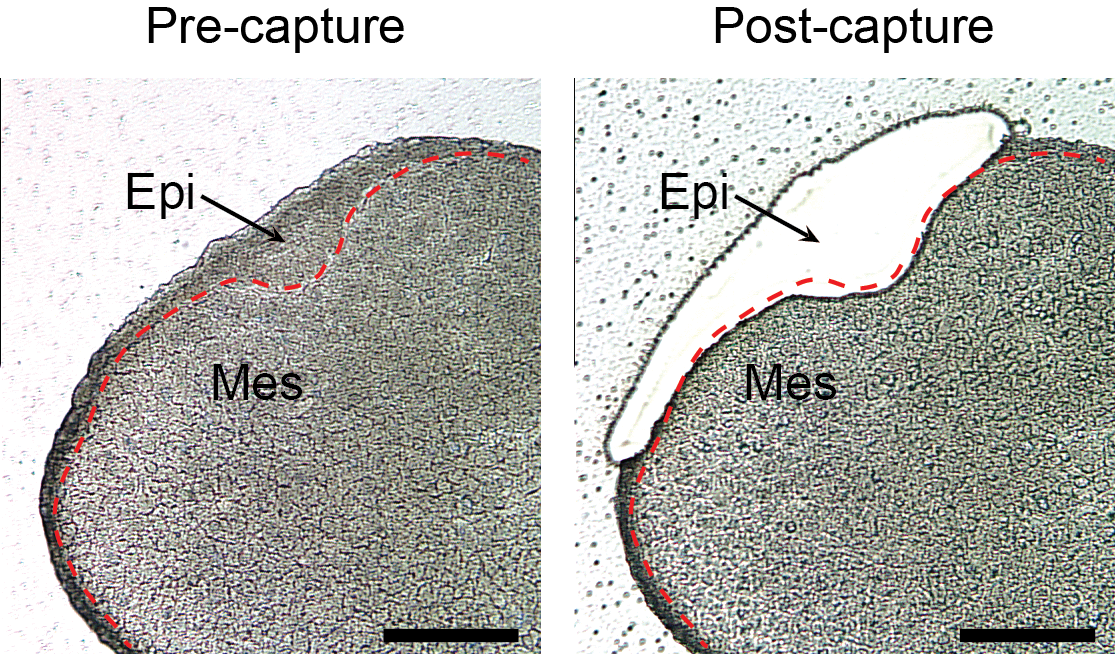

Supplement: Figure S3 — Laser capture microdissection (LCM) of epithelial incisor region. Sagittal sections of an E11.5 initiation stage incisor pre- and post-LCM. The captured epithelium was identified by bright field microscopy. Scale bar: 100 µm. (TIF) [file pone.0038568.s003.tif]

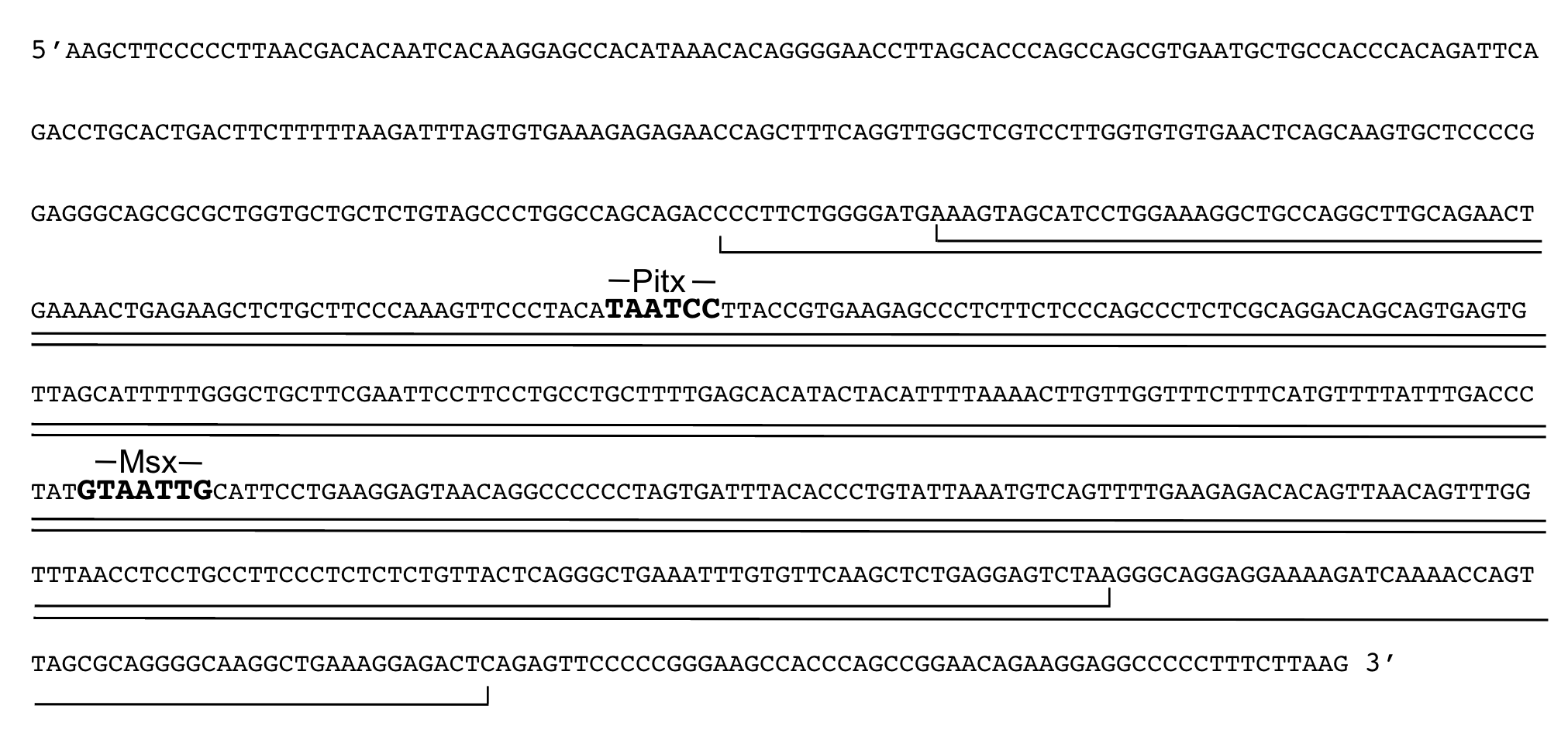

Supplement: Figure S4 — Pitx and Msx binding sites in the Bmp4 IE/LB enhancer. Sequence of the 758 bp region (chr14∶47,056,171–47,056,928) containing the 396 bp Bmp4 minimal enhancer double underlined (chr14∶47,056,283–47,056,678). The Pitx1/2 binding site (5′-TAATCC-3′) and Msx1/2 binding site (5′-GTAATTG-3′) are indicated, and the location of the 467 bp enhancer (chr14∶47,056,226–47,056,692) described by Chandler and coworkers [1] containing the 396 bp minimal enhancer sequence contained is underlined. (TIF) [file pone.0038568.s004.tif]

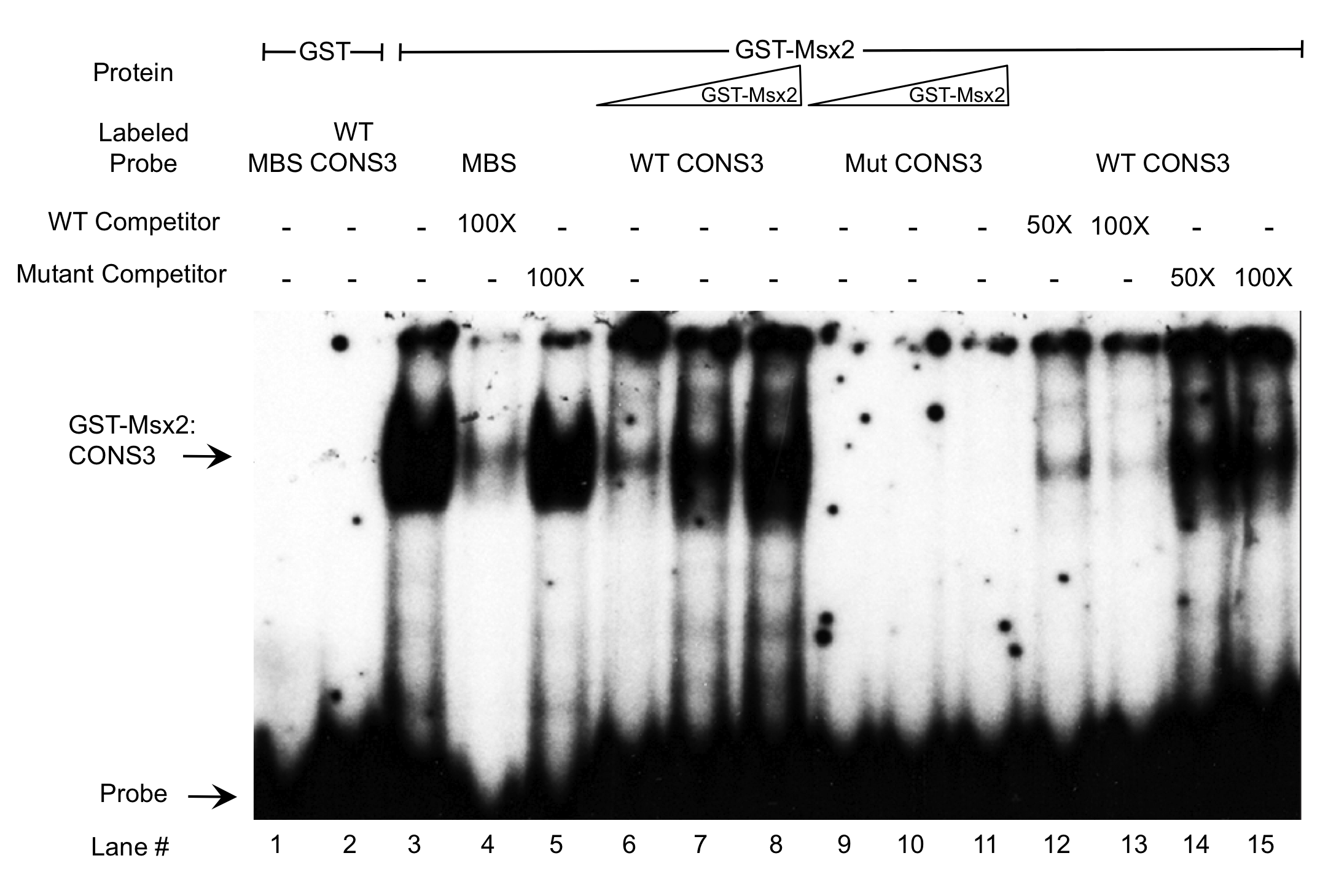

Supplement: Figure S5 — In vitro binding of Msx to the minimal Bmp4 enhancer. Electrophoretic Mobility Shift Assay (EMSA) exhibits robust binding of Msx2 protein to both a positive control MBS DNA sequence and to the consensus Msx1/2 binding site in the CONS3 sequence. Competition with specific or non-specific cold probes indicates sequence-specific binding of GST-Msx2 fusion protein to the consensus Msx1/2-binding motif. (TIF) [file pone.0038568.s005.tif]

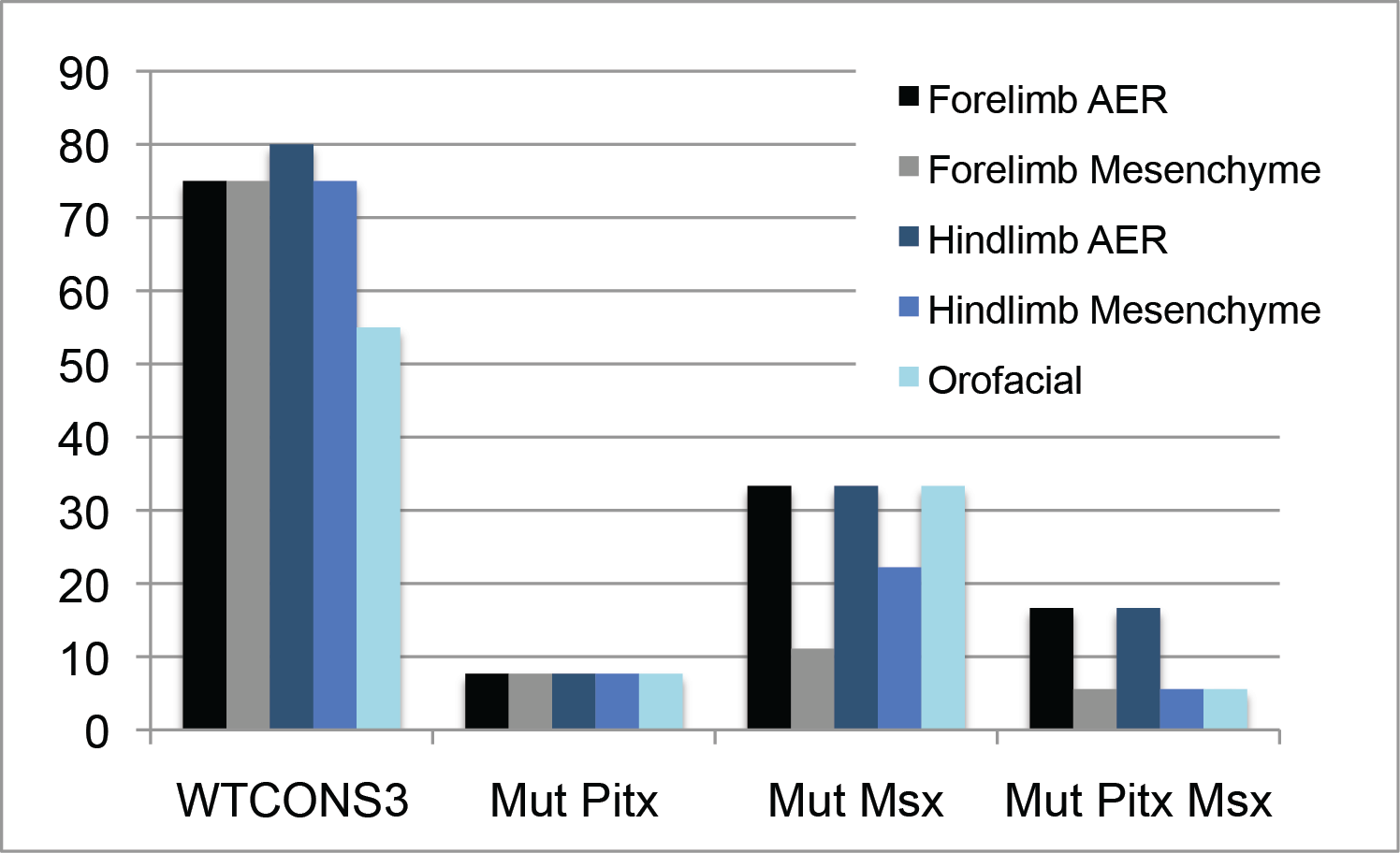

Supplement: Figure S6 — Transgenic mutational analysis of Pitx and Msx binding sites. Graphical representation of the transgenic mutational results presented in Fig. 5B indicates that Pitx binding site is necessary for reporter expression in forelimb and hindlimb AER and mesenchyme and orofacial tissues. (TIF) [file pone.0038568.s006.tif]

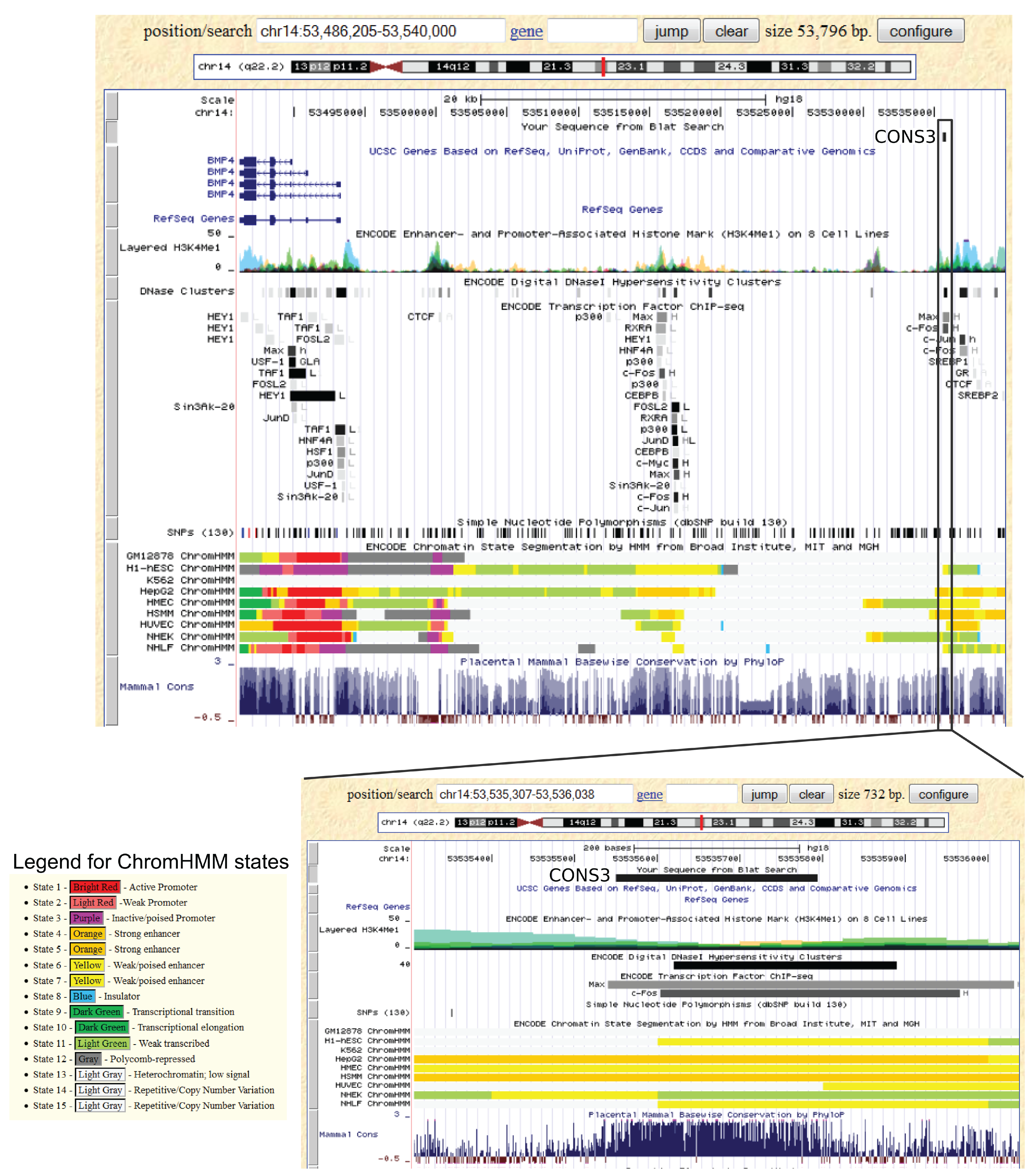

Supplement: Figure S7 — Integrative epigenomic analysis suggests that the human orthologous sequence of mouse CONS3 likely functions as an enhancer. Using public epigenomic data from the University of California at Santa Cruz (UCSC) Genome Browser, we analyzed the human homolog of CONS3 (labeled CONS3 at the upper right hand corner of the top genome browser view; genome assembly hg18). This region is enriched for an enhancer associated histone mark H3K4me1, is DNaseI hypersensitive, is a binding site for transcription factors Max and c-Fox in human embryonic stem cells, and is annotated to be in “enhancer state” in multiple human cell lines by ChromHMM [62]. These multiple lines of evidence suggest CONS3 homolog may also function as an enhancer in human. (TIF) [file pone.0038568.s007.tif]
