# Supplementary material for: An Evolutionarily Conserved Enhancer Regulates Bmp4 Expression in Developing Incisor and Limb Bud
Source: PLoS One. 2012 Jun 12;7(6):e38568. doi: 10.1371/journal.pone.0038568 (PMC3373496; doi:10.1371/journal.pone.0038568)
Supplement: Table S1 — Families of Transcription Factors (TFs) with their InterPro (IPR) DNA binding domain annotation number that have potential binding sequences in the minimal Bmp4 enhancer. Transcription factors families with binding sites (BS) in the minimal Bmp4 enhancer in >75% and >90% of aligned vertebrates are indicated. (PDF) [file pone.0038568.s008.pdf]

Table S1. Families of Transcription Factors (TFs) with their InterPro (IPR) DNA binding domain annotation number that have potential binding sequences in the minimal *Bmp4* enhancer.

E > 0.45			
**Binding Site (BS) in > 75% of aligned vertebrates**		**BS in > 90% of aligned vertebrates**	
TF	IPR Number	TF	IPR Number
DLX	IPR000047;IPR001356;IPR009057	DLX	IPR000047;IPR001356;IPR009057
ELF	IPR000418;IPR011991	HOXA	IPR001356;IPR009057
FOXJ	IPR001766;IPR011991	IRX	IPR001356;IPR009057
HBP	IPR009071	LEF	IPR009071
HIC	IPR007087;IPR015880	LHX	IPR001356;IPR009057
HOXA	IPR001356;IPR009057	MEIS	IPR001356;IPR009057
IRF	IPR001346;IPR011991	MSX	IPR000047;IPR001356;IPR009057
IRX	IPR001356;IPR009057	NRF	IPR001628
ISL	IPR001356;IPR009057	PITX	IPR001356;IPR009057
KLF	IPR007087;IPR015880	PKNOX	IPR001356;IPR009057
LEF	IPR009071	PRRX	IPR000047;IPR001356;IPR009057
LHX	IPR001356;IPR009057	PRRX	IPR001356;IPR009057
MEIS	IPR001356;IPR009057	TCFL	IPR009071
MSX	IPR000047;IPR001356;IPR009057	TGIF	IPR001356;IPR009057
MYBL	IPR001005;IPR009057		
NRF	IPR001628		
PITX	IPR001356;IPR009057		
PKNOX	IPR001356;IPR009057		
POUF	IPR000327;IPR000972;IPR001356;IPR009057;IPR010982;IPR013847		
POUF	IPR000327;IPR001356;IPR009057;IPR010982;IPR013847		
PRRX	IPR000047;IPR001356;IPR009057		
PRRX	IPR001356;IPR009057		
SIX	IPR000047;IPR001356;IPR009057		
SMAD	IPR001132;IPR003619;IPR013019		
SOX	IPR009071		
TCF	IPR009071		
TCF	IPR011598		
TCFL	IPR009071		
TGIF	IPR001356;IPR009057		
